# Icariin-loaded GelMa hydrogel encapsulated potassium sodium niobate biomimetic piezoelectric scaffold regulates macrophage polarization to accelerate bone defect repair

**DOI:** 10.1016/j.mtbio.2025.102476

**Published:** 2025-10-30

**Authors:** Yongbin Wang, Han Zhang, Zhili Xu, Weihang Zhu, Sheng Chang, Jiahao Wei, Shuqing Chen, Yong Liu, Weiqing Kong, Jianwei Guo

**Affiliations:** a Department of Orthopedics, The Affiliated Hospital of Qingdao University, No. 59, Haier Road, Laoshan District, Qingdao, 266003, People's Republic of China; b School of Anesthesiology, Shandong Second Medical University, No.7166, Baotong West Street, Weicheng District, Weifang, Shandong, 261053, People's Republic of China; c Department of Orthopedic Surgery, Xuzhou Central Hospital, Southeast University, Xuzhou Clinical School of Xuzhou Medical University, China

**Keywords:** Bone defect repair, 3D printing, Icariin, Macrophage polarization, Piezoelectric biological scaffolds, Immune microenvironment

## Abstract

Large bone defects present significant clinical challenges due to limitations of current treatments including immune rejection, infection, and poor osteoinductive capacity. This study developed a novel biomimetic piezoelectric scaffold system combining 3D-printed potassium sodium niobate/nano-hydroxyapatite/polylactic acid (KNN/nHA/PLA) scaffolds with icariin-loaded gelatin methacryloyl hydrogel (ICA@G/NHP) to synergistically promote bone regeneration and immunomodulation. The composite scaffolds demonstrated excellent mechanical properties and stable piezoelectric output under ultrasonic activation. In vitro studies revealed that ultrasonic stimulation was essential for activating piezoelectric effects, significantly enhancing osteogenic differentiation of bone marrow mesenchymal stem cells through upregulation of ALP, RUNX2, and COL1 expression. The incorporated icariin effectively promoted endothelial cell migration and induced M2 macrophage polarization via C-type lectin receptor signaling pathway, creating a pro-regenerative immune microenvironment. In vivo validation using rat cranial and rabbit femoral condyle defect models demonstrated superior bone regeneration with enhanced bone mineral density, bone volume fraction, and mature trabecular architecture compared to controls. Immunofluorescence analysis confirmed sustained M2 macrophage dominance and suppressed inflammatory responses. RNA sequencing identified PI3K/Akt as the central mechanotransduction pathway mediating scaffold effects. This integrated platform addresses dual challenges of osteogenesis and immune regulation, offering a promising therapeutic strategy for critical-sized bone defect repair through ultrasound-activated piezoelectric stimulation combined with targeted immunomodulation.

## Highlights

• A 3D-printed piezoelectric scaffold integrates with a drug-loaded hydrogel for enhanced bone regeneration.
• The scaffold promotes osteogenesis and regulates anti-inflammatory M2 macrophage polarization.
• Ultrasound-activated piezoelectricity boosts osteogenic markers 2.4-2.9 fold without cytotoxicity.
• Sustained icariin release ensures prolonged therapy throughout bone healing.
• In vivo results show significant bone repair with enhanced M2 infiltration and new bone formation.

## Graphical abstract

**Figure:**
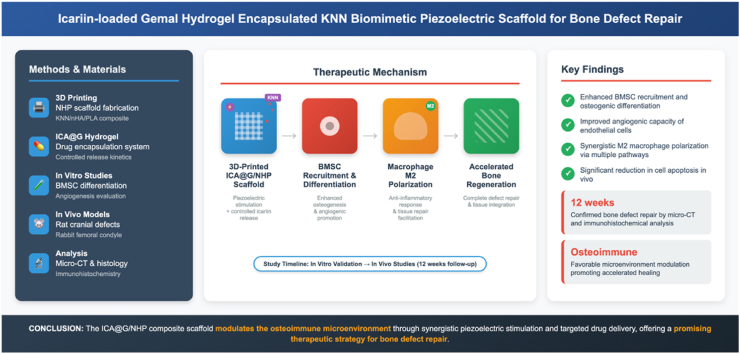

## 1 Introduction

Large bone defects caused by tumor resection, congenital anomalies, and trauma remain a complex clinical challenge. Allogeneic bone grafting is currently the most widely used clinical treatment [[1], [2], [3]].However, several factors limit its application, including immune rejection, disease transmission, infection, inflammation, and poor osteoinductive capacity [4,5].Compounding this issue is the global aging population, which has led to a severe shortage of allogeneic bone grafts that falls far short of clinical demands [6]. Furthermore, bioactive materials designed for bone defect repair in tissue engineering often induce inflammatory responses, resulting in necrosis within the implanted bone repair materials [7,8].Therefore, it is imperative to prioritize the development of bioactive materials capable of effectively promoting bone regeneration by modulating immune microenvironmental mechanisms.These clinical limitations underscore the urgent need for innovative biomaterial strategies that can simultaneously promote bone regeneration while actively modulating the immune microenvironment to prevent inflammatory complications. Bone tissue engineering has emerged as a promising approach for bone repair and regeneration [9,10].To address the dual challenges of osteogenesis and immune regulation, the development of bioactive scaffolds that effectively modulate cellular behaviors—such as proliferation, migration, and differentiation—represents a critical component in this field [11,12].The bioelectrical phenomena naturally occurring in bone tissue have been widely recognized for their significant roles in skeletal development and fracture healing [13,14]. Furthermore, endogenous electrical signals have been demonstrated to regulate macrophage behaviors, including migration, phagocytic activity, and cytokine production [[15], [16], [17]].The potential mechanisms through which electrical stimulation promotes osteogenesis include upregulation of intracellular Ca^2+^ concentrations associated with osteoblasts, primary opening of voltage-gated Ca^2+^ channels, and accelerated osteogenesis via the enhancement of calmodulin signaling pathways [18,19]. The concept of piezoelectric-induced osteogenesis has driven the development of piezoelectric implants for bone regeneration [20,21].Recent studies have demonstrated that surface charge polarization, electrical field stimulation, and the functional characteristics of piezoelectric biomaterials synergistically regulate cellular functions and tissue regeneration both in vitro and in vivo [22,23].Piezoelectric biomaterials, such as poly-L-lactic acid (PLA), nano-hydroxyapatite (nHA), and potassium sodium niobate (KNN), generate physiological electrical microenvironments that play crucial roles in enhancing metabolic activities [24,25]. Clinical electrostimulation therapies have already demonstrated the capacity to promote bone healing and spinal fusion. Additionally, modulation of immune cell fate and tissue regeneration has attracted considerable attention [26].External induction of macrophage polarization toward the anti-inflammatory M2 phenotype triggers selective expression and secretion of anti-inflammatory factors, regulating the local immune microenvironment and promoting bone tissue repair [[25], [26], [27]].Porous scaffolds represent promising bone graft materials as they provide cell-friendly microenvironments, mimic bone structure through 3D printing, and serve as templates that facilitate cellular interactions and formation of bone extracellular matrix, thereby promoting reparative osteogenesis [28] Polylactic acid (PLA) and nano-hydroxyapatite (nHA) exhibit excellent biosafety, biocompatibility, and low toxicity without antigenicity or immunogenicity, having received FDA approval as biomaterials for human medical applications. Synthetic PLA possesses predictable and reproducible mechanical and physical properties [29].Galassi et al. obtained high-density potassium sodium niobate (KNN) particles (>94 %) with excellent piezoelectric properties (d_33_ = 97 pC/N) through prolonged high-energy ball milling of pre- and post-calcination powders, with toxicological testing confirming the absence of cytotoxicity in KNN materials [30].Among various piezoelectric ceramics available for biomedical applications, potassium sodium niobate (KNN) presents distinct advantages that make it particularly suitable for bone tissue engineering applications [31].Unlike conventional lead-based piezoelectric materials such as lead zirconate titanate (PZT), which demonstrate superior piezoelectric coefficients but raise significant biocompatibility concerns due to lead toxicity and potential leaching in physiological environments, KNN offers a compelling lead-free alternative that addresses both performance and safety requirements [32].The lead-free nature of KNN eliminates the risk of heavy metal ion release, which could potentially cause cellular toxicity, inflammatory responses, and adverse systemic effects—critical considerations for long-term implantable devices [33].Furthermore, KNN exhibits exceptional biocompatibility profiles compared to other piezoelectric ceramics, with studies demonstrating minimal cytotoxic effects and excellent cellular tolerance in various biological systems [34].The material's chemical stability in aqueous environments, combined with its ability to maintain consistent piezoelectric properties under physiological conditions, positions KNN as an ideal candidate for sustained electrical stimulation in bone regeneration applications [35].Additionally, the piezoelectric coefficient of KNN (d_33_ = 97 pC/N) provides sufficient electrical output to generate physiologically relevant electrical fields that can effectively modulate cellular behaviors, including osteoblast proliferation, differentiation, and extracellular matrix synthesis, while maintaining the safety profile essential for clinical translation [35]. While piezoelectric materials offer promising solutions for electrical stimulation-mediated bone regeneration, the complex immune microenvironment surrounding bone defects necessitates additional therapeutic strategies beyond physical stimulation alone. This recognition has led researchers to explore the integration of bioactive compounds that can complement piezoelectric effects by directly targeting immune cell behavior and inflammatory responses. Building upon osteoconductive piezoelectric scaffolds, incorporating active compounds or molecules into composite materials presents significant advantages [36]. Epimedium, widely used in traditional Chinese medicine as a "kidney-tonifying herb," demonstrates potential for preventing osteoporosis and osteonecrosis [37,38].Icariin, the primary active component of Epimedium, is an 8-prenylated flavonol glycoside appearing as a pale yellow powder with the molecular formula C_33_H_40_O_15_ [39].Recent research has established icariin's definitive anti-osteoporotic effects, similar to estrogen, stimulating bone formation while inhibiting bone resorption [40].Importantly, beyond its direct osteogenic properties, both in vitro and in vivo data indicate that icariin and its metabolite (ICT) can modulate immune cell function and activation, regulate inflammatory factor release, and restore abnormal signaling pathways, such as macrophage polarization [41].This dual functionality—promoting osteogenesis while simultaneously modulating immune responses—makes icariin an ideal candidate for combination with piezoelectric materials to create synergistic therapeutic effects. Therefore, incorporating icariin into scaffolds to form porous composite materials represents a highly promising concept for developing biomaterials and potentially offers an approach to promote bone defect repair [42].

Macrophages, derived from bone marrow myeloid progenitor cells, constitute an essential component of the innate immune system [3].They play vital roles in inflammation, tissue repair, and metabolism. Monocyte-macrophages demonstrate diversity and plasticity, capable of differentiating into various phenotypes that fulfill different functions depending on the microenvironment—a process termed macrophage polarization—which plays crucial roles in numerous physiological and pathological processes [43].Research indicates that icariin can mediate M2-type polarization; M2 macrophages are characterized by high expression of scavenger molecules and possess efficient phagocytic and immunoregulatory functions that promote tissue repair [[44], [45], [46]]. The aggregation and differentiation of bone marrow mesenchymal stem cells (BMSCs) at bone defect sites, followed by bone and cartilage formation, are regulated by multiple factors in the local microenvironment [47].The interactions and precise regulation between macrophages and osteogenic cells play increasingly important roles in endochondral and intramembranous ossification processes. Inflammatory cytokines secreted by M2 macrophages can promote osteogenic differentiation of BMSCs [3].

Despite extensive research on piezoelectric materials for bone regeneration and icariin's immunomodulatory properties, previous studies have not explored the combined effects of KNN's piezoelectricity and icariin's immunomodulation within a single 3D-printed scaffold system. Current approaches typically focus on either physical stimulation through piezoelectric materials or biochemical modulation through bioactive compounds, but rarely integrate both strategies in a synergistic manner. This represents a significant gap in the field, as the complex nature of bone defect healing requires simultaneous regulation of multiple cellular processes including osteogenesis, angiogenesis, and immune response.

**Fig. 1:**
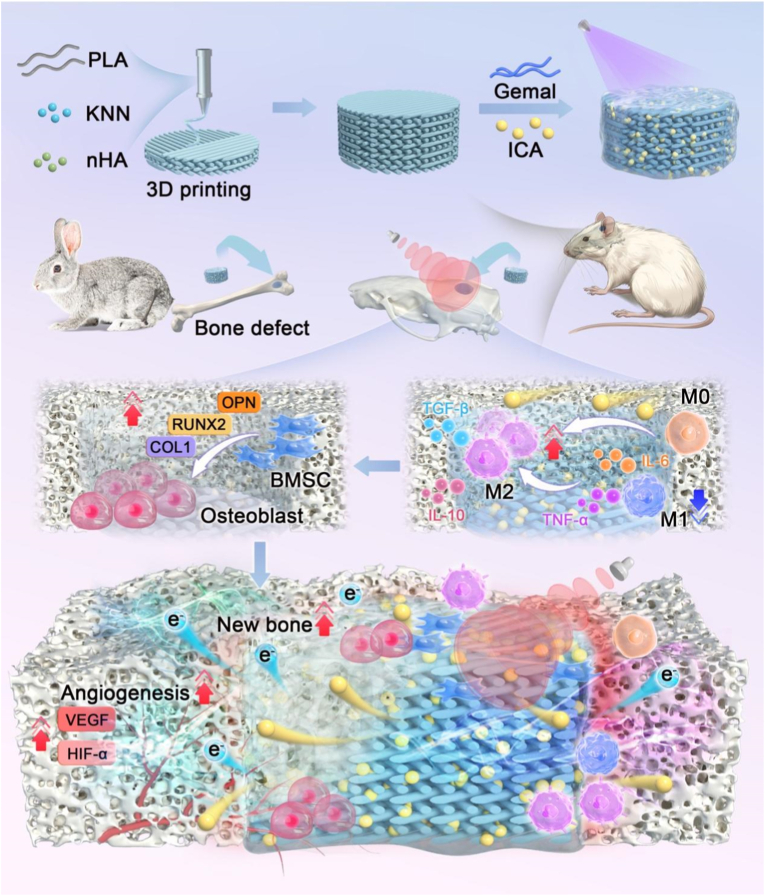
Schematic illustration demonstrates ICA-loaded 3D-printed NHP piezoelectric scaffolds modulating the immune microenvironment and enhancing angiogenesis to accelerate bone defect repair.

As illustrated in Fig. 1, our strategy combines KNN-induced electrical stimulation with icariin-mediated immunomodulation in a 3D-printed scaffold platform, offering an unprecedented opportunity to create a comprehensive therapeutic system that addresses the multifaceted challenges of large bone defect repair. In this study, we 3D-printed NHP piezoelectric scaffolds using KNN, nHA, and PLA, and infused them with a mixture of icariin (ICA) and GelMa hydrogel solution, subsequently crosslinked via UV photocuring [48].The physical and chemical properties were tested to verify that the enhanced piezoelectric characteristics resulted from KNN incorporation into the composite scaffold. Given that the KNN-based piezoelectric composite scaffolds can provide in situ stable electrical stimulation, we evaluated the practicality of ICA@G/NHP scaffolds in large-scale bone repair by systematically investigating their in vivo and in vitro healing-promoting capabilities, immunomodulatory functions, as well as angiogenic and osteogenic activities. Finally, RNA transcriptomic analysis was performed to validate the key signaling pathways through which the composite scaffolds regulate bone regeneration.

## 2 Experimental section

### 2.1 Materials

Niobium pentoxide (Nb_2_O_5_), potassium carbonate (K_2_CO_3_), sodium carbonate (Na_2_CO_3_), anhydrous ethanol (C_2_H_5_OH), polylactic acid ((C_3_H_4_O_2_)_n_), nano-hydroxyapatite (Ca_5_(PO_4_)_3_(OH)), icariin (C_33_H_40_O_15_), methanol (CH_3_OH), dexamethasone, L-ascorbic acid, and β-glycerophosphate disodium were purchased from Aladdin Reagent Co., Ltd. (Shanghai, China). Fetal bovine serum (FBS) and antibiotics (penicillin/streptomycin, PS) were obtained from Gibco BRL (Shanghai, China). Gelatin methacryloyl (GelMA) was provided by EFL Co., Ltd. (Suzhou, China).

### 2.2 Preparation of KNN powder

Potassium sodium niobate powder was prepared using a conventional solid-phase sintering method. Niobium pentoxide (34.262g), potassium carbonate (6.831g), and sodium carbonate (8.907g) were weighed according to the specified dosages. The three reagents were then mixed with 200 mL of anhydrous ethanol and ball-milled for 8 h to obtain a premix. The ball-milled mixture was stirred on a magnetic stirrer for 2h, dried, and then calcined in a high-temperature box furnace at 750 °C for 2 h to produce potassium sodium niobate powder.

### 2.3 3D printing of NHP scaffolds

NHP scaffolds were fabricated using a biomedical-grade 3D printing system (BIO-EM3DP15A, Qingdao Five-Dimensional Intelligent Additive Manufacturing Technology Co., Ltd.) via a fused deposition modeling (FDM) process. For NHP material preparation, polylactic acid (PLA) and nano-hydroxyapatite (nHA) were mixed at a mass ratio of 1:4, with potassium sodium niobate (KNN) added at concentrations of 0, 5, 10, and 15 wt%, respectively. The mixture was compounded using a torque rheometer at 180°Cfor 30 min to ensure homogeneous distribution.During FDM printing, the material barrel and nozzle were preheated to 190 °C and 200 °C, respectively, while the printing platform was maintained at 60 °C. After thermal equilibration for 15 min, the NHP filament was loaded into the printer under controlled backpressure of 380 ± 30 kPa. The printing parameters were optimized as follows: nozzle height of 0.1 mm, layer thickness of 0.2 mm, and printing speed of 12 mm/s.Following scaffold fabrication, the entire assembly was subjected to a polarization treatment by heating to 120 °C and applying an electric field intensity of 3.0 kV/mm for 30 min. The system was then cooled to 25 °C at a controlled rate of 1.5 °C/min before removing the electric field. This polarization process was essential for activating the piezoelectric properties of the KNN particles within the composite matrix.

### 2.4 Assembly of KNN/nHA/PLA/ICA@G scaffolds (ICA@G/NHP)

The voids in the NHP scaffolds were filled with GelMA hydrogel containing icariin (ICA) to enhance the scaffold's ability to modulate the immune microenvironment. Briefly, the ICA solution was diluted to the target concentration. Subsequently, 8 % GelMA was mixed with the ICA solution at a 1:1 ratio to prepare the ICA@G solution, ensuring a final ICA concentration of 10 μM. The solution was drawn into a syringe and directly injected into pre-sterilized scaffolds, followed by in situ crosslinking using 405 nm blue light. Through this method, the final ICA@G/NHP integrated scaffolds were formed.

### 2.5 Characterization of assembled scaffolds

The surface morphology and microstructure of the assembled scaffolds were observed using a digital camera and scanning electron microscope (SEM, GAIA3, Czechoslovakia). Additionally, the mechanical strength of PG, PT, and PT/G scaffolds was evaluated using a universal material testing machine (AG-10KNIS, Japan) at a constant loading rate of 0.1 cm/min. The compressive strength of 1 cubic centimeter cubes was also assessed. Each test was conducted in triplicate. The distribution of key elements in the piezoelectric hydrogel was scanned using energy dispersive spectroscopy (UltimMax 40, Oxford, UK). Functional group changes in the piezoelectric hydrogel were detected via X-ray diffraction (XRD; Rigaku, Japan) and Fourier transform infrared spectroscopy (FTIR; TNZ1-5700, Nicolet, USA). The output voltage of the piezoelectric hydrogel was determined using a digital storage oscilloscope (RTM3000, Rohde & Schwarz, Germany).

### 2.6 Mechanical property evaluation

The mechanical properties of the scaffolds were comprehensively evaluated through multiple testing methods. Static compression testing was performed using a universal material testing machine (AG-10KNIS, Japan) on cylindrical specimens (diameter: 5 mm, height: 4 mm) at a constant loading rate of 0.1 cm/min until failure. The compressive strength of cubic specimens (1 cm^3^) was also assessed under identical conditions. Each test was conducted in triplicate (n = 3) to ensure data reliability.To assess the evolution of mechanical properties during scaffold degradation, systematic in vitro evaluation was conducted based on modified protocols from ASTM D5511 and ISO 23317 standards. Cylindrical scaffold specimens (diameter: 5 mm, height: 4 mm) were prepared for each scaffold type (HP, NHP, and ICA@G/NHP) using the aforementioned optimized 3D printing parameters. For each scaffold composition, specimens were divided into multiple groups corresponding to different immersion time points: 0 days (dry control group), 5 days, 10 days, 15 days, 25 days, and 40 days (n = 6 for each group at each time point). Initial dimensions were precisely measured using digital calipers to ensure consistency in stress calculations. Specimens were immersed in revised simulated body fluid (r-SBF) prepared according to the Kokubo protocol, with ionic concentrations matching human plasma (Na^+^: 142.0 mM, K^+^: 5.0 mM, Mg^2+^: 1.5 mM, Ca^2+^: 2.5 mM, Cl^−^: 147.8 mM, HCO_3_^−^: 4.2 mM, HPO_4_^2−^: 1.0 mM, SO_4_^2−^: 0.5 mM). Immersion was conducted in an incubator maintained at 37 ± 0.5 °C with 5 % CO_2_ atmosphere to maintain physiological pH (7.4 ± 0.2). The solution-to-specimen volume ratio was maintained at 30:1 (mL:mg) to prevent saturation effects, and r-SBF solution was completely replaced every 3 days to maintain stable ionic concentrations and prevent degradation product accumulation. At predetermined time points, specimens were carefully removed from r-SBF solution, gently rinsed with distilled water to remove surface salt deposits, and briefly blotted with filter paper to remove excess moisture while maintaining hydrated state. Compression testing was immediately performed using a universal testing machine (Instron 5969, USA) equipped with a 10 kN load cell at a crosshead speed of 1 mm/min until specimen failure. Testing was conducted under ambient conditions to simulate clinical handling situations, with testing completed within 5 min of specimen removal to minimize dehydration effects. Compressive strength (σc) and elastic modulus (E) were calculated from stress-strain curves. Strength retention was calculated as: Strength retention (%) = (σc,t/σc,0) × 100 %, where σc,t represents the compressive strength at time t and σc,0 represents the initial compressive strength. Similarly, modulus retention was calculated as: Modulus retention (%) = (Et/E0) × 100 %. Statistical analysis was performed using one-way analysis of variance (ANOVA) followed by Tukey's post hoc test, with p < 0.05 considered statistically significant.

### 2.7 Fatigue performance testing

To evaluate the long-term mechanical stability of the scaffolds under physiological loading conditions, cyclic compressive fatigue testing was performed to obtain stress-life (S-N) curves. Cylindrical fatigue test specimens (diameter: 5 mm, height: 4 mm) were fabricated for each scaffold type (HP, NHP, and ICA@G/NHP) using the aforementioned optimized 3D printing parameters. Fatigue testing was conducted on a universal testing machine (Instron 5969, USA) equipped with a 10 kN load cell and fatigue testing module. Testing was performed under stress-controlled mode, applying sinusoidal cyclic loading at a frequency of 2 Hz to simulate the physiological loading frequency of human daily activities, with a stress ratio (R = σmin/σmax) set to 0.1 to mimic the cyclic loading characteristics experienced by bone tissue in vivo. Testing was conducted at different maximum stress levels, ranging from 50 %, 60 %, 70 %, to 80 % of the ultimate compressive strength for each material to establish complete S-N relationship curves. Six parallel specimens (n = 6) were tested at each stress level to ensure data reliability. Testing continued until specimen fatigue failure (defined as load-bearing capacity decreasing to below 25 % of maximum load) or reaching a predetermined cycle limit (10^7^ cycles, defined as fatigue limit). Testing was performed at room temperature with relative humidity controlled at 45–55 % to simulate clinical environmental conditions. Fatigue life was defined as the number of cycles experienced from the start of loading until failure occurred. S-N curves were plotted using double logarithmic coordinates, with the horizontal axis representing the number of cycles (N) and the vertical axis representing the maximum stress level (S).

### 2.8 Preparation of extracts from scaffolds

Extract solutions from the experimental groups were prepared according to previously reported protocols. The scaffolds were immersed in FBS-free culture medium (α-MEM or DMEM) at a concentration of 20 mg/mL and then incubated at 37 °C for 24 h.

### 2.9 Cell Isolation and culture

BMSCs were extracted from the bone marrow cavities of four-week-old SD rats according to established literature methods. The specific process was as follows: experimental animals were euthanized by cervical dislocation, soaked in 75 % ethanol for 5 min for whole-body disinfection, and then bilateral femurs and tibias were aseptically isolated in a clean bench. Surrounding soft tissues were removed, and the bones were repeatedly washed three times with PBS containing antibiotics. The bone ends were then cut to expose the bone marrow cavity, and pre-cooled α-MEM culture medium was injected into the bone marrow cavity using a 23G needle syringe, repeatedly flushing until the bone tissue appeared transparent white. The bone marrow suspension was collected and filtered through a 70 μm cell strainer to remove bone fragments and cell clumps. The filtrate was centrifuged at 1500×*g* for 10 min, and the pellet was resuspended in complete α-MEM culture medium containing 10 % FBS and 1 % penicillin-streptomycin. Cells were seeded at a density of 5 × 10^5^/cm^2^ onto tissue culture (TC) treated culture dishes and maintained under strictly controlled conditions: 37 ± 0.5 °C, 5 ± 0.2 % CO_2_ atmosphere, and 95 ± 3 % relative humidity in a humidified CO_2_ incubator (Thermo Scientific Heracell VIOS 160i). The culture medium pH was maintained at 7.2–7.4, and dissolved oxygen levels were monitored to remain above 18 %. The first media change was performed exactly 72 h after seeding to remove non-adherent cells, followed by complete media changes every 48–72 h (specifically every 2–3 days) with fresh pre-warmed culture medium. Medium changes were performed at consistent intervals to maintain optimal nutrient levels and prevent metabolic waste accumulation. When cell confluence reached 70–80 % (previously 80 %), 0.25 % trypsin-EDTA was used for 1–3 min (previously 2-min) digestion at 37 °C, and cells were passaged at a 1:3 ratio. All culture medium was pre-equilibrated to 37 °C and pH 7.2–7.4 before use. Cells from passages P3-P5 were used for experiments to ensure proliferative activity and stem cell characteristics.Cell viability was assessed using trypan blue exclusion assay, maintaining >95 % viability throughout all passages.Cell identification was performed by flow cytometry detection of CD90/CD29 positive rates (>95 %) and CD34/CD45 negative rates (>99 %), and multipotential differentiation was verified through osteogenic (Alizarin Red staining) and adipogenic induction (Oil Red O staining). All cell culture procedures were conducted in a Class II biological safety cabinet under sterile conditions. The entire process complied with animal experimental ethics standards, and operation temperatures were strictly controlled at 37 ± 1 °C to maintain cell viability, with all solutions pre-warmed and incubation time outside the controlled environment minimized to less than 10 min per handling session.

### 2.10 Biocompatibility assessment of scaffolds

According to literature methods, the specific process for biocompatibility assessment experiments was as follows: BMSCs and HUVECs were co-cultured with UV-sterilized scaffold materials in 24-well plates at a density of 1 × 10^4 cells per well. The scaffolds were pre-soaked in α-MEM/DMEM culture medium for 2 h to balance surface charges. The co-culture system contained 1 mL of mixed culture medium with 10 % FBS per well and was cultured at 37 °C with 5 % CO_2_. For cell proliferation detection, CCK-8 reagent (diluted at a 9:1 ratio with culture medium) was used on days 1, 3, and 5, with light-protected incubation for 2 h. To avoid adsorption of formazan products by the scaffold materials, the supernatant was transferred to a 96-well plate before measuring absorbance at 450 nm. For cell viability analysis, Calcein-AM/PI double staining was performed after 24/72 h of co-culture using 600 μL of working solution (2 μM Calcein-AM + 4 μM PI) at room temperature for 15 min. The distribution of live cells (green) and dead cells (red) was observed using a fluorescence microscope. For apoptosis detection, co-cultured cells from 6-well plates were collected using trypsin without EDTA, washed with pre-cooled PBS, and resuspended in 1 × binding buffer. After Annexin V-FITC and 7-AAD double staining (light-protected incubation for 15 min), the proportions of early/late apoptosis were analyzed by flow cytometry. For in vivo assessment, tissues were collected on days 6/12 post-scaffold implantation, fixed in 4 % neutral formaldehyde, embedded in paraffin, and sectioned at 5 μm. H&E staining was used to observe inflammatory cell infiltration and new blood vessel formation, and tissue compatibility indices were quantitatively analyzed using ImageJ software. Throughout the experiment, blank scaffold groups, cell-only groups, and positive control groups (containing cytotoxic substances) were established. All data were verified through three independent experiments.

### 2.11 Transwell migration assay

To evaluate the effects of the experimental groups on the in vitro migration abilities of BMSCs and HUVECs, this study used a Transwell migration experimental system (24-well plate, 8 μm pore size polycarbonate membrane) for quantitative analysis. The experimental and control group scaffolds were pre-equilibrated with 600 μL of culture medium containing specific treatments and placed in the lower chamber of the Transwell. The upper chamber was seeded with 200 μL of serum-free α-MEM containing BMSCs suspension (2 × 10^4^ cells/well) or D-MEM containing HUVECs suspension (2 × 10^4^ cells/well), respectively. The system was then incubated at 37 °C with 5 % CO_2_ for 24 h. After incubation, sterile cotton swabs were used to gently remove non-migrated cells from the upper surface of the membrane. Cells that had migrated to the bottom of the membrane were fixed with 4 % paraformaldehyde for 30 min (room temperature), stained with 1 % crystal violet for 10 min, and rinsed with PBS to remove residual dye before air-drying. Migrated cells were imaged under an inverted optical microscope (Nikon Eclipse Ti2, Japan) at 100 × magnification. ImageJ software was used to count cells in randomly selected five fields, and the data were normalized for comparative analysis with the control group. This experimental design strictly followed the principles of methodological reproducibility by clearly specifying membrane pore size parameters, standardizing culture conditions (temperature, CO_2_ concentration), and equipment model details (such as microscope, software version) to ensure result objectivity. The staining and fixation steps were optimized to effectively reduce background interference, and the quantitative analysis strategy (random field sampling and data normalization) further enhanced statistical power, providing reliable support for subsequent mechanism research.

### 2.12 In vitro osteogenic differentiation

This study employed a co-culture system of scaffold materials and bone marrow mesenchymal stem cells (BMSCs) for osteogenic differentiation evaluation. The specific experimental steps were as follows: BMSCs were seeded at a density of 1 × 10^4 cells/well onto the surface of scaffold materials pre-placed in 24-well plates and cultured under standard conditions at 37 °C with 5 % CO_2_. After 24 h, the medium was changed to osteogenic induction medium (Cyagen Biosciences, China), and fresh induction medium was changed at 08:00 and 16:00 daily thereafter to maintain a stable induction microenvironment. On day 7 of induction culture, alkaline phosphatase (ALP) activity detection and staining analysis were performed. After PBS rinsing, samples were fixed with 4 % paraformaldehyde (Solarbio, China) at room temperature for 15 min, followed by cell chemical staining using Evensen's modified method (ALP staining kit, ELK Biotechnology, China). For quantitative detection, cells were treated with RIPA lysis buffer (containing 1 % protease inhibitor), centrifuged at 12,000×*g* for 10 min, and the supernatant was collected for colorimetric analysis using the pNPP chromogenic system (ALP activity detection kit, Beyotime, China). A standard curve was established using p-nitrophenol standards at gradient concentrations, and the absorbance values of all samples and control groups were measured at 405 nm using a NEO2SALPHA enzyme-labeled instrument (BioTek, USA). To systematically evaluate late-stage mineralized nodule formation, Alizarin Red S (ARS) staining was added on day 21 of induction culture. After PBS washing, samples were fixed with pre-cooled 95 % ethanol for 15 min, stained with 0.5 % ARS solution (pH 4.2) at room temperature in the dark for 20 min, and repeatedly washed with ultrapure water to remove non-specific staining before observing calcium salt deposition under an inverted optical microscope (IX83, Olympus, Japan). Quantitative analysis used 10 % hexadecylpyridinium chloride solution for desorption, measuring absorbance at 562 nm and converting to calcium salt deposition amount according to the standard curve. All experiments were set up with three biological replicates, and statistical analysis was performed using GraphPad Prism 9.0 software for one-way analysis of variance (One-way ANOVA). Data were expressed as mean ± standard deviation, with p < 0.05 considered statistically significant.

### 2.13 In vitro HUVEC migration

To evaluate the effect of scaffold extracts on the migration ability of BMSCs/HUVECs, this study used a scratch wound healing assay for analysis. HUVECs were seeded at a density of 5 × 10^4^ cells/well in 24-well plates and cultured to approximately 90 % confluence. A standardized scratch was then created on the monolayer using a sterile 200 μL pipette tip, followed by three PBS washes to remove detached cells and debris, and subsequent replacement with culture medium containing scaffold extracts. Cell migration was observed using an inverted microscope, and images were captured at 0 h and 24 h after scratching. Cell migration ability was quantified by measuring the change in scratch width at each time point, using the following formula:

$$Relative\;wound\;closure\;\left(\%\right)=\left(1-\frac{W_{t}}{W_{0}}\right)\times 100$$

Image J software was used to measure scratch width, and experimental data were expressed as mean ± standard deviation (mean ± SD) from three independent repeated experiments. This method optimized the traditional area analysis method to a precise distance-based measurement method, significantly improving the accuracy of cell migration assessment.

### 2.14 Quantitative assessment of In vitro HUVECs angiogenic capacity

This study evaluated the in vitro angiogenic capacity of Human Umbilical Vein Endothelial Cells (HUVECs) using a Matrigel tube formation experimental system. The specific experimental procedure was as follows: First, according to BD Company (USA) product instructions, 100 μL of Matrigel was used to pre-coat 48-well plate wells and solidified at 37 °C for 30 min to form a three-dimensional growth substrate. Subsequently, HUVECs were seeded at a density of 30,000 cells/well in fresh DMEM culture medium pre-incubated for 1 h (37 °C, 5 % CO_2_), while the biomaterial scaffolds to be tested were placed in the upper layer of a Transwell chamber (pore size 0.4 μm) to construct a co-culture system. After continuous induction for 6 h under standard culture conditions (37 °C, 5 % CO_2_), cells were fixed with 4 % paraformaldehyde at room temperature for 15 min to stabilize the tubular structures. Microscopic images of the tubular networks formed on the Matrigel surface were captured using a Leica inverted optical microscope (CKX53, Germany), with five random fields (10 × objective) selected for standardized imaging for each sample. Finally, quantitative assessment of angiogenesis-related parameters was performed based on the morphometric analysis module of Image J software (developed by NIH), including the number of branch points, mesh count per unit area, and the length of the longest master segment, thereby characterizing the tube formation potential of HUVECs and the pro-angiogenic effect of the scaffold materials.

### 2.15 Real-Time Quantitative Polymerase Chain Reaction (RT-qPCR)

Real-Time Quantitative Polymerase Chain Reaction (RT-qPCR): Gene expression related to osteogenic differentiation (ALP, Runx2, COL1, OPN) and angiogenesis (vWF, CD31, ANG) was detected by RT-qPCR(Table S1–S3, Supporting Information). The expression of macrophage polarization markers (TNF-α, iNOS, Arg1, CD206) was also evaluated.Housekeeping genes β-actin and GAPDH were used as internal references for normalization. Cells were first digested with trypsin, and total RNA was extracted using Trizol reagent (Shanghai Sangon Biotech, China). The mRNA was then reverse transcribed to cDNA using the Prime Script RT reagent kit (Takara, Japan). Subsequently, RT-PCR reactions were performed using the SYBR Green RT-PCR kit (Biomake, USA) and a real-time PCR system (Thermo Fisher, USA), with all operations conducted according to the manufacturer's protocols. Results normalized to the control group.

### 2.16 Western blot analysis

After cell culture, treatment with extracts from different scaffolds was performed, followed by Western blot analysis of protein expression levels. Proteins were extracted using RIPA lysis buffer provided by Beyotime Company (China), and protein concentrations were quantified using the BCA protein detection kit produced by the same company. Protein samples were separated by sodium dodecyl sulfate-polyacrylamide gel electrophoresis (SDS-PAGE) provided by ELK Biotechnology Company (China) and then transferred to polyvinylidene difluoride (PVDF) membranes produced by Millipore Company (China). Subsequently, the membranes were incubated overnight at 4 °C with specific primary antibodies, thoroughly washed with TBST buffer, and then incubated with corresponding secondary antibodies at room temperature for 1 h. Finally, ECL reagent from Shanghai Share-bio Company (China) was used for color detection, and Image J software was used for quantitative analysis of protein band intensity.

### 2.17 Sprague-Dawley rat surgical procedure

To evaluate the bone regeneration ability of ICA@G/NHP, the study established a critical-sized calvarial defect model using 8-week-old male Sprague-Dawley rats (weighing 200–250 g). The animals were divided into four experimental groups: the control group received no treatment after the calvarial defect model was established; the ICA@G group received a one-time in situ injection with crosslinking of GelMA and ICA mixture (10 μM) under 405 nm blue light for 30 s; the NHP and NHP/ICA@G groups received their respective scaffold implants. During the surgical procedure, rats were anesthetized by intraperitoneal injection of 1 % pentobarbital sodium (3.5 mg/100 g), the scalp was longitudinally incised to expose the calvarium, and bilateral parietal defects were created using a 5 mm drill bit. The incision was sutured post-operation, and animals were allowed free access to food and water. Rats were sacrificed at 6- and 12-weeks post-surgery, and calvarial tissues were collected and fixed in 4 % paraformaldehyde. Three-dimensional reconstruction was performed using micro-computed tomography (micro-CT), and CT-Analyser software was used to analyze bone mineral density (BMD), bone volume fraction (BV/TV), trabecular separation (Tb.Sp), and trabecular thickness (Tb. Th). For histological analysis, specimens were decalcified in 20 % EDTA for 6 weeks, and 5 μm sections were prepared for H&E, Masson, and Picrosirius Red (PSR) staining. At 12 weeks post-surgery, immunohistochemistry was used to assess new bone formation using anti-bone sialoprotein (BSP) and anti-osteocalcin (OCN) primary antibodies (Servicebio, Wuhan, China; 1:200 dilution) incubated overnight at 4 °C, and rabbit IgG secondary antibodies (2-step plus Poly-HRP Anti Rabbit IgG Detection System) incubated at room temperature for 2 h.

### 2.18 Adult New Zealand white rabbit surgical procedure

To evaluate the bone regeneration ability of ICA@G/NHP in a rabbit femoral condyle defect model, the study established a critical-sized femoral condyle defect model using adult New Zealand white rabbits (weighing 2.5–3.0 kg). The experimental animals were randomly divided into four groups: the control group received no treatment after the femoral condyle defect model was established; the ICA@G group received a one-time in situ injection with crosslinking of GelMA and ICA mixture (10 μM) under 405 nm blue light for 30 s; the NHP and NHP/ICA@G groups received their respective scaffold implants. During the surgical procedure, rabbits were anesthetized by ear marginal vein injection of 3 % pentobarbital sodium (1.0 mL/kg), and the knee joint area was shaved and disinfected to expose it. A longitudinal incision was made at the lateral femoral condyle, and a cylindrical defect with a diameter of 5 mm and a depth of 10 mm was created using a specialized drill bit. After implanting the corresponding materials, the incision was sutured in layers, and antibiotics were administered post-surgery to prevent infection. Animals were allowed free movement and access to food and water post-surgery. Experimental animals were sacrificed at 6 and 12 weeks post-surgery, and femoral condyle tissues were collected and fixed in 4 % paraformaldehyde. Three-dimensional reconstruction was performed using micro-computed tomography (micro-CT), and CT-Analyser software was used to analyze bone mineral density (BMD), bone volume fraction (BV/TV), trabecular separation (Tb.Sp), and trabecular thickness (Tb.Th). For histological analysis, specimens were decalcified in 20 % EDTA for 6 weeks, and 5 μm sections were prepared for H&E and Masson staining. At 12 weeks post-surgery, immunofluorescence was used to assess new bone formation.

### 2.19 microRNA sequencing and Bioinformatics analysis

To explore changes in microRNA expression profiles during ICA@G/NHP-induced osteogenic differentiation of BMSCs, total RNA was extracted from BMSCs in the ICA@G/NHP treatment group and the control group. Total cellular RNA was extracted using TRIzol reagent (Invitrogen, USA) according to the manufacturer's instructions, and RNA concentration and purity were detected using a NanoDrop 2000 spectrophotometer (Thermo Fisher Scientific, USA). RNA integrity was assessed using an Agilent 2100 Bioanalyzer (Agilent Technologies, USA). Qualified RNA samples were then sent to KangChen Biotech Co., Ltd. (China) for small RNA library construction and high-throughput sequencing. Library preparation was conducted using the NEBNext® Multiplex Small RNA Library Prep Set for Illumina® (New England BioLabs, USA), strictly following standard procedures. Briefly, 3 μg of total RNA was used as starting material, first connecting 3′ and 5′ adapters, then synthesizing cDNA first strand by reverse transcription, followed by PCR amplification. PCR products were separated by 6 % polyacrylamide gel electrophoresis, and bands of approximately 140–160 bp (containing microRNA insert fragments and adapter sequences) were cut out, recovered, and purified to construct the small RNA library. The Illumina HiSeq platform (Illumina, USA) was used for single-end 50 bp sequencing. After quality control processing of the sequencing data, Cutadapt software (v1.18) was used to remove adapter sequences and low-quality read segments to obtain clean reads. Clean reads were compared with the human genome (GRCh38/hg38), and reads were mapped to the known miRBase (v22.0) human microRNA database using Bowtie software (v1.3.0). The DESeq2 software package (v1.30.1) was used to identify differentially expressed microRNAs between the ICA@G/NHP treatment group and the control group, with criteria set at |log2(fold change)|≥1 and adjusted p-value<0.05. Target genes of differentially expressed microRNAs were predicted using miRWalk, TargetScan, miRDB, and miRTarBase databases, with only target genes predicted by at least two databases selected for subsequent analysis. GO functional enrichment and KEGG pathway analysis of the predicted target genes were performed using the DAVID online tool (v6.8) and Metascape platform. Additionally, a microRNA-mRNA interaction network diagram was constructed to visualize key regulatory relationships, with visualization performed using Cytoscape software (v3.8.2). Selected differentially expressed microRNAs were verified by qRT-PCR to confirm the reliability of the sequencing results.

### 2.20 Statistical analysis

Continuous variables are presented as mean ± standard deviation (SD). For multiple comparison analyses, one-way analysis of variance with Tukey's post-hoc test was employed. Statistical evaluations were performed using GraphPad Prism software (GraphPad Software Inc., USA). The significance level for tests was set at ∗p < 0.05, ∗∗p < 0.01, ∗∗∗p < 0.001, indicating progressively increasing levels of statistical correlation.

## 3 Results and discussion

### 3.1 3D printing, characterization, and optimization of NHP scaffolds

Three-dimensional (3D) printing has been widely applied in bone tissue engineering for manufacturing scaffolds with customizable shapes and internal structures. Polylactic Acid (PLA) is biodegradable and non-toxic, making it widely used in tissue engineering. However, PLA's hydrophobicity and lack of surface cell recognition sites limit its application in bone tissue engineering. Hydroxyapatite (HA), composed of calcium phosphate, is the main inorganic component of natural bone. However, HA's lack of toughness, elasticity, and poor bone induction ability makes it difficult to process, greatly limiting its applications. Combining inorganic components such as strontium, magnesium, and zinc with organic polymers has proven to be an effective method to improve osteogenesis and bone induction ability, promoting bone tissue regeneration. Recent studies have demonstrated that surface charge polarization or electrical field stimulation, as well as the functional characteristics of piezoelectric biomaterials like potassium sodium niobate (KNN) and barium titanate (BaTiO_3_), synergistically regulate cell function or tissue regeneration in vitro/in vivo. Beyond the chemical composition of scaffolds, the availability of cell attachment sites, adequate biomechanical properties, the ability to participate in bone remodeling processes, and porous structures suitable for cell migration and proliferation are also essential characteristics of osteogenic scaffolds. Previous research has confirmed that the HA content in scaffolds varies within the range of 0–30 %. When the mass ratio exceeds 30 %, the ink cannot melt and extrude, making it unsuitable for 3D printing. In this study, cylindrical NHP scaffolds (diameter 5 mm, thickness 400 μm) with different KNN mass ratios were prepared using fused deposition modeling-based 3D printing technology. First, CCK-8 assays on days 1, 3, and 5 indicated that NHP scaffolds could support the attachment and proliferation of bone marrow mesenchymal stem cells. The NHP scaffold group with 10 %Wt KNN maintained the strongest proliferation of bone marrow mesenchymal stem cells (Fig. S1). Bone marrow mesenchymal stem cells (BMSCs) were seeded onto NHP scaffolds to assess cell adhesion. On days 7 and 14 post-seeding, cells spread along the fibers and exhibited an elongated morphology without a specific alignment pattern (Fig. S2).

**Fig. 2:**
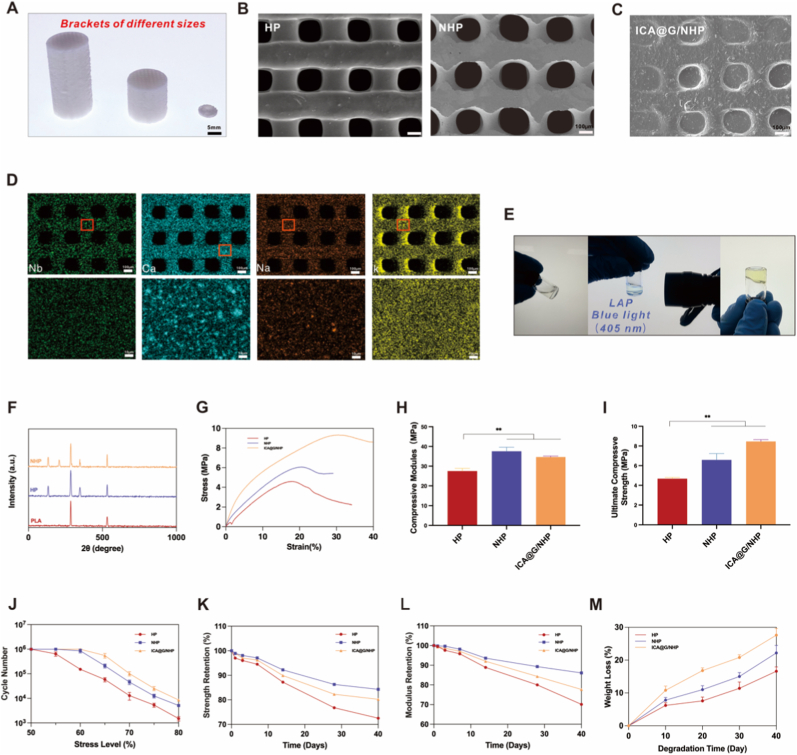
Fabrication, characterization, and performance evaluation of 3D-printed composite scaffolds. (A)Photographs of NHP scaffolds with different dimensions fabricated via 3D printing technology, demonstrating scalability and printing precision. (B) Scanning electron microscopy images of HP and NHP scaffolds showing regularly interconnected and ordered porous structures. The gradient microchannels between PLA fibers provide sufficient mechanical performance and smaller chambers conducive to osteoblast differentiation, while maximizing diffusion of nutrients and oxygen for better microvascular growth. (C) SEM image of ICA@G/NHP composite scaffold showing successful infiltration of ICA-loaded GelMA hydrogel into the porous network and formation of stable structure through photocrosslinking, maintaining original porosity while providing enhanced bioactivity and cell recognition sites. (D)Energy dispersive X-ray spectroscopy elemental mapping of NHP scaffolds, confirming that the four main elements—Nb, Ca, P, and O—are uniformly distributed in PLA, validating the multi-level compositional characteristics of the hybrid scaffolds. (E) Photographic documentation of ICA@G hydrogel photocuring process using 405 nm blue light (intensity: 10 mW/cm^2^, duration: 20s) to rapidly crosslink the ICA@G prepolymer into a stable hydrogel network. (F) X-ray diffraction patterns revealing differentiated elemental characteristics of various scaffold types. Pure PLA scaffolds primarily show characteristic peaks of carbon (C, 285.0 eV) and oxygen (O, 532.0 eV); HP composite scaffolds detect additional characteristic peaks of calcium (Ca, 347.0 eV) and phosphorus (P, 133.0 eV), confirming successful introduction of nano-hydroxyapatite; NHP composite scaffolds further display significant signals of potassium (K, 293.0 eV) and niobium (Nb, 207.0 eV). (G) Stress-strain curves from uniaxial compression testing, showing all formulations demonstrate elastic-plastic deformation characteristics typical of porous biomaterials. (H) Quantitative comparison of compressive modulus, with HP scaffolds at 28.5 ± 2.1 MPa, KNN incorporation significantly enhancing mechanical properties with NHP scaffolds reaching 38.2 ± 2.8 MPa, and ICA@G/NHP composite scaffolds further improving to 35.6 ± 2.9 MPa. (I) Ultimate compressive strength comparison showing HP scaffolds at 4.8 ± 0.3 MPa, NHP scaffolds achieving 6.5 ± 0.4 MPa (35 % and 34 % improvements respectively), and ICA@G/NHP composite scaffolds reaching 8.2 ± 0.5 MPa. These mechanical properties approach those of cancellous bone (2–12 MPa compressive strength). (J) Stress-life fatigue curves under simulated physiological loading conditions, tested on a universal testing machine applying sinusoidal cyclic loading at 2 Hz frequency with stress ratio of 0.1. ICA@G/NHP composite scaffolds exhibit optimal fatigue performance with fatigue life exceeding 5 × 10^6^ cycles at 50 % stress level. (K) Compressive strength retention monitoring during 40-day degradation. HP scaffolds retain 96.8 ± 2.1 % after 5 days and decline to 72.5 ± 3.8 % after 40 days; NHP scaffolds show 92.8 ± 2.9 % and 84.2 ± 4.2 % respectively; ICA@G/NHP scaffolds exhibit 89.6 ± 3.1 % and 75.8 ± 4.5 %. (L) Compressive modulus retention evolution, with all scaffold formulations maintaining >85 % mechanical integrity during the critical early healing period (0–15 days). (M) Mass loss analysis in revised simulated body fluid. HP scaffolds show the slowest degradation rate with 16.02 % mass loss after 40 days; KNN incorporation in NHP scaffolds significantly accelerates degradation, achieving 22.3 %; ICA@G/NHP composite scaffolds exhibit the most rapid degradation at 26.66 %, with controlled degradation profiles well-matched to physiological bone regeneration timelines.

The research team prepared NHP scaffolds with different thicknesses, line diameters, and diameters to verify the printability of these scaffolds (Fig. 2A).Scanning electron microscopy (SEM) images (Fig. 2B) showed that the scaffolds possessed regularly interconnected and ordered porous structures with pore sizes of 200 μm. The gradient microchannels between PLA fibers would provide sufficient mechanical performance and smaller chambers conducive to osteoblast differentiation, as well as maximize the diffusion of nutrients and oxygen for better microvascular growth. Subsequently, energy dispersive spectrometry (EDS) spectra and X-ray diffraction (XRD) confirmed that the four main elements in NHP—Nb, Ca, P, and O—were present and uniformly distributed in PLA(Fig. 2D and F). XRD analysis clearly revealed the differentiated elemental characteristics of various scaffold types. Pure PLA scaffolds primarily showed characteristic peaks of carbon (C, 285.0 eV) and oxygen (O, 532.0 eV), consistent with their polymer matrix characteristics. In HP composite scaffolds, additional characteristic peaks of calcium (Ca, 347.0 eV) and phosphorus (P, 133.0 eV) were detected, confirming the successful introduction of nano-hydroxyapatite (nHA). Notably, NHP composite scaffolds further displayed significant signals of potassium (K, 293.0 eV) and niobium (Nb, 207.0 eV), jointly verifying the integration of potassium sodium niobate (KNN) in the composite structure. The above results systematically support the multi-level compositional characteristics of the hybrid scaffolds. Based on the above mechanical and biological analyses, we used NHP scaffolds with 30 % nHA and 10 %Wt KNN for subsequent studies.

### 3.2 Fabrication of ICA@G/NHP composite scaffolds

To enhance the bioactivity and osteogenic potential of the 3D-printed NHP scaffolds, a functionalized hydrogel system was developed and integrated with the scaffolds. GelMA hydrogel was selected due to its excellent biocompatibility, tunable physicochemical properties, and ability to mimic the natural extracellular matrix (ECM) microenvironment. The high water content (>90 %) and three-dimensional porous structure of GelMA effectively support cell migration, proliferation, and nutrient transport.Icariin (ICA), a flavonoid compound with established osteogenic activity, was incorporated into the GelMA hydrogel to construct the ICA@G system. Based on preliminary CCK-8 assays with bone marrow mesenchymal stem cells (BMSCs) treated with various ICA concentrations (0, 10, 50, and 90 μM), 50 μM was determined as the optimal concentration, showing the highest cell viability and proliferation (Fig. S3).The ICA@G/NHP composite scaffolds were fabricated through the following procedure: First, the NHP scaffolds were 3D-printed using the previously described method and sterilized under UV light. The ICA@G prepolymer solution was prepared by dissolving GelMA (10 % w/v) in PBS at 60 °C, followed by the addition of ICA (50 μM) and photoinitiator LAP (0.25 % w/v). The pre-warmed ICA@G solution was then carefully infiltrated into the porous structure of the NHP scaffolds using a pipette, ensuring complete penetration into the scaffold pores through gentle vacuum assistance. The infiltrated scaffolds were subsequently exposed to 405 nm blue light (intensity: 10 mW/cm^2^, duration: 20s) to rapidly cross-link the ICA@G prepolymer into a stable hydrogel network within the scaffold architecture (Fig. 2E). This photocuring process resulted in a homogeneous composite structure where the ICA@G hydrogel was uniformly distributed throughout the NHP scaffold, maintaining the original porosity while providing enhanced bioactivity and cell recognition sites.

### 3.3 Characterization of ICA@G/NHP scaffolds

Microstructural and chemical characterization confirmed successful integration of the ICA@G hydrogel within the NHP scaffold architecture. Scanning electron microscopy revealed that the ICA@G hydrogel was uniformly incorporated into the interconnected porous network while preserving the original architectural integrity (Fig. 2C). The composite scaffolds maintained a highly ordered macroporous structure with the hydrogel forming uniform coatings on scaffold strut surfaces and filling microporous regions without occluding macropores, creating a hierarchical porous structure that combines mechanical stability with bioactive functionality. High-magnification images demonstrated intimate contact between the crosslinked hydrogel network and scaffold substrate, indicating successful photopolymerization without delamination or phase separation. Fourier transform infrared spectroscopy confirmed the preservation of all component characteristics without significant peak shifts, indicating physical compatibility rather than chemical reaction (Fig. S4). The FTIR spectrum showed characteristic KNN peaks at 680 cm^−1^ and 850 cm^−1^ (Nb-O vibrations), PLA ester carbonyl stretching at 1750 cm^−1^, hydroxyapatite phosphate vibrations at 1000-1100 cm^−1^, GelMA amide bands at 1650 cm^−1^ and 1540 cm^−1^, and icariin characteristic peaks at 1605 cm^−1^, 1260 cm^−1^, and 1070 cm^−1^, confirming uniform drug distribution within the hydrogel network.Mechanical testing revealed progressive property enhancement across scaffold formulations under uniaxial compression (Fig. 2G–I). HP scaffolds exhibited baseline performance with compressive strength of 4.8 ± 0.3 MPa and modulus of 28.5 ± 2.1 MPa. KNN incorporation significantly enhanced mechanical properties, with NHP scaffolds achieving 6.5 ± 0.4 MPa compressive strength and 38.2 ± 2.8 MPa modulus (35 % and 34 % improvements, respectively) due to ceramic particle reinforcement and improved stress transfer efficiency. ICA@G/NHP composite scaffolds demonstrated further enhancement, reaching 8.2 ± 0.5 MPa compressive strength and 35.6 ± 2.9 MPa modulus, approaching cancellous bone properties (2–12 MPa) while avoiding stress-shielding effects. The crosslinked hydrogel network contributed to mechanical enhancement by filling void spaces and providing additional load-bearing pathways. Cyclic fatigue testing under physiological conditions (2 Hz, stress ratio 0.1, up to 10^7^ cycles) revealed exceptional performance, with ICA@G/NHP scaffolds withstanding >5 × 10^6^ cycles at 50 % stress level and ∼8 × 10^4^ cycles at 80 % stress level (Fig. 2J). This superior fatigue resistance was attributed to the crack-arresting and stress-dispersing effects of the hydrogel component through viscoelastic damping properties.Degradation studies in simulated body fluid over 40 days revealed controlled, composition-dependent kinetics well-matched to bone regeneration timelines (Fig. 2M). HP scaffolds showed the slowest degradation (16.02 % mass loss), while KNN incorporation accelerated degradation in NHP scaffolds (22.3 % mass loss) due to ionic release creating localized pH fluctuations that enhance PLA hydrolytic cleavage. ICA@G/NHP composites exhibited the most rapid degradation (26.66 % mass loss) through synergistic effects of hydrogel water content, enzymatic susceptibility, and amino acid fragment catalysis. Mechanical integrity monitoring throughout degradation demonstrated that all formulations maintained >85 % strength during the critical early healing period (0–15 days), providing essential structural support during initial inflammatory and proliferative phases (Fig. 2K and L). The more rapid mechanical degradation of ICA@G/NHP scaffolds in later stages (15–40 days) aligns favorably with natural bone remodeling timelines, facilitating gradual load transfer to newly formed bone tissue while preventing stress-shielding effects that could impair bone maturation.

### 3.4 Piezoelectric properties of the composite scaffold

**Fig. 3:**
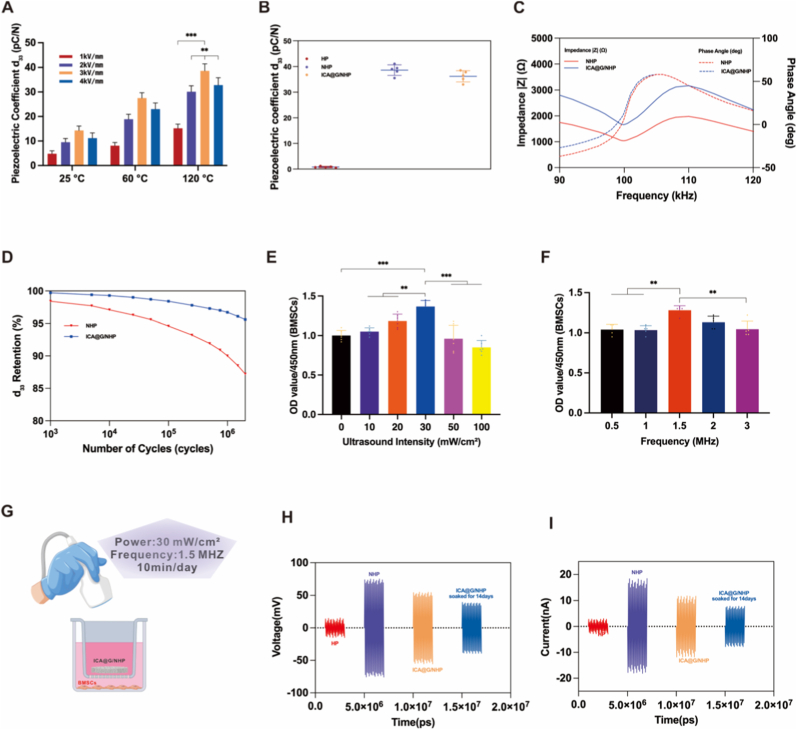
Piezoelectric characterization and LIPUS activation of composite scaffolds. (A) Optimization results of poling process showing strong dependence of piezoelectric coefficient (d_33_) on electric field strength (1.0–4.0 kV/mm) and temperature (25 °C, 60 °C, 120 °C). At room temperature (25 °C), d_33_ values remained low (<15 pC/N) even at high fields (4.0 kV/mm); optimal performance was achieved at 120 °C under 3.0 kV/mm for 30 min, resulting in maximum d_33_ value of 38.6 ± 2.8 pC/N. (B) Quantitative evaluation of piezoelectric coefficient of scaffolds poled under optimal conditions. HP scaffold lacking piezoelectric KNN phase exhibited negligible d_33_ value (0.8 ± 0.3 pC/N); NHP scaffold achieved higher d_33_ of 38.6 ± 2.8 pC/N; ICA@G/NHP scaffold resulted in comparable d_33_ value (36.2 ± 3.1 pC/N), indicating functionalization process well preserved piezoelectric functionality. (C) Frequency response characterized by impedance spectroscopy Bode plots revealing characteristic resonance peaks for both groups. Significant increase in overall impedance magnitude was observed for ICA@G/NHP scaffold indicating damping effect introduced by hydrogel matrix, while resonance frequency remained identical at 99.6 kHz. (D) Evaluation under simulated physiological conditions (37 °C Hank's solution) using MTS dynamic fatigue testing system. Specimens underwent 2 × 10^6^ cycles of sinusoidal loading at 1 Hz with stress range of 2–8 MPa, with NHP and ICA@G/NHP scaffolds exhibiting retention rates of d_33_ piezoelectric coefficient reaching 95.6 % and 87.2 % respectively. (E) CCK-8 assay results of BMSCs under different ultrasound intensities (0–100 mW/cm^2^), with 30 mW/cm^2^ intensity showing optimal cell viability reaching 135 % of unstimulated controls by day 3. (F) CCK-8 assay results of BMSCs under different ultrasound frequencies (0.5–3.0 MHz), with 1.5 MHz frequency showing optimal cell viability reaching 125 % of unstimulated controls by day 3. (G) Schematic illustration of LIPUS experimental setup with parameters of power 30 mW/cm^2^, frequency 1.5 MHz, 10 min daily stimulation. (H)Voltage-time profiles under LIPUS activation. HP control scaffold exhibited voltage fluctuations limited to ±8–10 mV; NHP scaffold generated substantial alternating voltage signals with peak amplitudes reaching ±75–80 mV; ICA@G/NHP scaffold demonstrated comparable voltage generation (±70–75 mV) and retained significant piezoelectric activity with ±45–50 mV after 14 days of physiological soaking, representing approximately 65 % retention of initial response. (I)Corresponding current-time measurements corroborating voltage response patterns. HP scaffold showed negligible current generation (±1–2 nA); NHP scaffold produced significant alternating currents with peak amplitudes of ±18–20 pA; ICA@G/NHP scaffold maintained similar current output (±16–18 nA) and demonstrated ±10–12 nA current generation after 14 days of physiological conditioning, corresponding to approximately 60–65 % retention of initial response.

The piezoelectric functionality of the scaffolds was activated by electrical poling. To determine the optimal conditions, we systematically evaluated the effects of temperature and electric field strength on the piezoelectric coefficient (d_33_) (Fig. 3A). This process revealed strong dependencies; room temperature poling was ineffective (d_33_ < 15 pC/N), while elevated temperatures enhanced domain mobility. Consequently, optimal poling was achieved at 120 °C with a 3.0 kV/mm field for 30 min [49], parameters exceeding KNN's coercive field (∼1.0–1.5 kV/mm) to ensure full polarization.Quantitative measurements confirmed that the significant piezoelectric response (d_33_ = 38.6 ± 2.8 pC/N for NHP; d_33_ = 36.2 ± 3.1 pC/N for ICA@G/NHP) originated from the polarized KNN, as the HP control scaffold without KNN showed only baseline noise (d_33_ = 0.8 ± 0.3 pC/N) (Fig. 3B). The achieved output is within the range known to elicit beneficial biological responses, such as enhanced osteogenesis and immunomodulation [50]. The scaffolds' mechanical stability was evaluated under physiological conditions. Impedance spectroscopy showed a characteristic resonance at 99.6 kHz for both NHP and ICA@G/NHP scaffolds (Fig. 3C). The hydrogel incorporation increased overall impedance due to damping but preserved piezoelectric behavior. Dynamic fatigue testing (37 °C, Hank's solution, 2 × 10^6^ cycles, 1 Hz, 2–8 MPa) demonstrated excellent durability, with d_33_ retention rates of 95.6 % (NHP) and 87.2 % (ICA@G/NHP) (Fig. 3D). These values exceed those of conventional piezoelectric ceramics (<80 % retention). The superior performance is likely due to the composite structure: the PLA matrix mitigates stress, nHA enhances toughness, and the hydrogel homogenizes the electric field. The fatigue behavior followed a predictable power-law relationship (∝σ^0.6, R^2^ = 0.96, p < 0.01), aiding clinical prediction. For non-load-bearing applications (e.g., cranial repair), we used low-intensity pulsed ultrasound (LIPUS) to activate the piezoelectric response. The parameters (1.5 MHz, 30 mW/cm^2^, 10 min/day) were selected based on established therapeutic protocols [[51], [52], [53]]. BMSCs cultured under this regimen showed enhanced metabolic activity, confirming biocompatibility (Fig. 3E–G). Real-time characterization confirmed robust activation: NHP scaffolds generated substantial voltages (±75–80 mV) and currents (±18–20 nA) under LIPUS, while HP controls showed minimal response (±8–10 mV, ±1–2 nA) (Fig. 3H and I).ICA@G/NHP scaffolds maintained comparable initial performance (±70–75 mV, ±16–18 nA) with excellent stability after 14-day physiological conditioning, retaining approximately 65 % voltage output and 60–65 % current generation. This LIPUS-piezoelectric coupling mechanism involves acoustic wave conversion to mechanical strain within KNN crystallites, generating alternating electrical polarization that creates biomimetic electrical stimulation mirroring endogenous bioelectrical phenomena during bone remodeling. This non-invasive platform enables precise control of the bioelectrical microenvironment. Its stable, biomimetic electrical output effectively promotes osteogenesis and immunomodulation, ensuring sustained bioactivity for enhanced bone regeneration.

### 3.5 In vitro biocompatibility of composite scaffolds

**Fig. 4:**
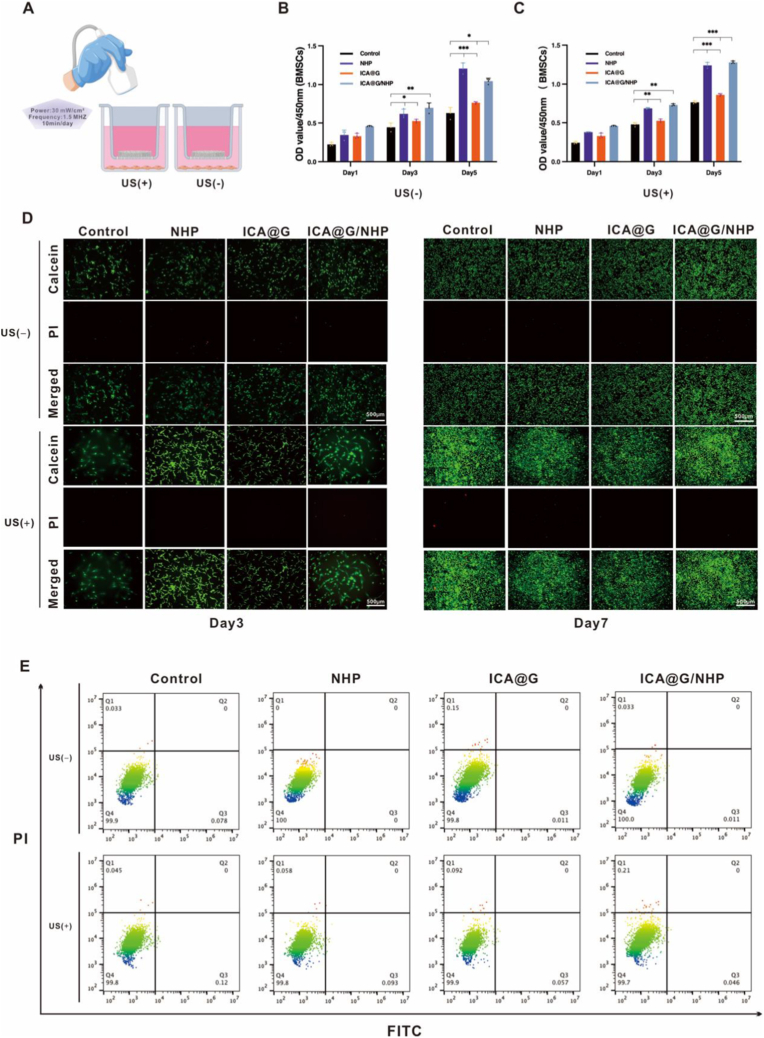
In vitro biocompatibility assessment of composite scaffolds. (A) Schematic illustration of the experimental setup showing ultrasound stimulation protocols with US(−) representing static culture conditions and US(+) representing low-intensity pulsed ultrasound stimulation (1.5 MHz, 30 mW/cm^2^, 10 min/day). (B, C) CCK-8 proliferation assay results showing optical density values at 450 nm over 5 days of culture under static conditions (B) and ultrasonic stimulation conditions (C). Data represent mean ± SD (n = 6). Statistical significance: ∗∗p < 0.01, ∗∗∗p < 0.001 compared to control group; no significant differences observed between US(−) and US(+) conditions within the same group (p > 0.05). (D) Live/dead fluorescence staining images showing cell viability on different scaffold surfaces at day 3 and day 7. Green fluorescence (calcein-AM) indicates live cells, red fluorescence (propidium iodide) indicates dead cells. (E) Flow cytometric apoptosis analysis using FITC-Annexin V/PI double staining. The plots show cell population distributions in four quadrants: Q1 (necrotic cells, FITC-/PI+), Q2 (late apoptotic cells, FITC+/PI+), Q3 (early apoptotic cells, FITC+/PI-), and Q4 (viable cells, FITC-/PI-). Numbers in each quadrant represent the percentage of cells in that population.

The biocompatibility of the composite scaffolds represents a fundamental prerequisite for their clinical translation in bone tissue engineering applications. To comprehensively evaluate the cellular responses to the different scaffold formulations, a systematic in vitro assessment was conducted using bone marrow mesenchymal stem cells (BMSCs) as the representative cell model. The biocompatibility evaluation encompassed multiple complementary assays including metabolic activity assessment, live/dead viability staining, and apoptosis analysis under both static and ultrasonic stimulation conditions.Cell metabolic activity and proliferation were quantitatively assessed using the CCK-8 (Cell Counting Kit-8) assay over a 5-day culture period. Under static culture conditions (US-), all scaffold groups demonstrated excellent biocompatibility with progressive increases in optical density values, indicating robust cellular proliferation and metabolic activity (Fig. 4B). The control group (tissue culture plastic) served as the baseline, showing steady proliferation kinetics with OD values of 0.31 ± 0.02, 0.45 ± 0.03, and 0.62 ± 0.04 on days 1, 3, and 5, respectively. The NHP scaffold group exhibited comparable proliferation patterns with OD values of 0.29 ± 0.02, 0.41 ± 0.03, and 0.58 ± 0.03, demonstrating that the incorporation of KNN and nHA components did not induce cytotoxic effects. Notably, both the ICA@G group (0.33 ± 0.02, 0.49 ± 0.03, 0.69 ± 0.04) and the ICA@G/NHP composite group (0.35 ± 0.02, 0.52 ± 0.04, 0.71 ± 0.05) showed enhanced proliferation compared to control and NHP groups, particularly evident from day 3 onwards. This enhancement can be attributed to the bioactive properties of icariin, which promotes cellular metabolism and proliferation through multiple signaling pathways.Importantly, the application of low-intensity pulsed ultrasound stimulation (US+) did not significantly alter cellular proliferation patterns across all groups (Fig. 4C). Under ultrasonic stimulation, the proliferation kinetics remained essentially unchanged compared to static conditions. The control group achieved OD values of 0.30 ± 0.02, 0.44 ± 0.03, and 0.61 ± 0.04 on days 1, 3, and 5, showing no statistically significant difference from static culture conditions (p > 0.05). Similarly, the NHP scaffold group maintained comparable OD values of 0.28 ± 0.02, 0.40 ± 0.03, and 0.57 ± 0.03, and the ICA@G/NHP composite group exhibited OD values of 0.34 ± 0.03, 0.51 ± 0.04, and 0.70 ± 0.05. These results demonstrate that the ultrasonic stimulation protocol (1.5 MHz, 30 mW/cm^2^, 10 min/day) does not interfere with normal cellular metabolic processes or induce stress responses, confirming the biocompatibility and safety of the stimulation parameters for long-term therapeutic applications.Live/dead viability staining provided direct visualization of cellular survival and distribution on the scaffold surfaces (Fig. 4D). Fluorescent microscopy images revealed predominantly green fluorescence (calcein-AM, live cells) with minimal red fluorescence (propidium iodide, dead cells) across all experimental groups at both day 3 and day 7 time points. Under both static culture conditions (US-) and ultrasonic stimulation conditions (US+), all groups demonstrated excellent cell viability with dense cellular populations and uniform distribution throughout the scaffold surfaces. The cells exhibited typical spread morphology with extensive cytoplasmic extensions, indicating strong cell-substrate adhesion and normal cellular behavior. Critically, no discernible differences in cell density, distribution patterns, or morphology were observed between static and ultrasonic stimulation conditions, further confirming that the ultrasound protocol does not adversely affect cellular behavior or viability. Quantitative analysis of the live/dead ratio consistently exceeded 95 % across all groups and conditions, confirming the excellent biocompatibility of all scaffold formulations and the absence of cytotoxic effects from both material components and ultrasonic stimulation protocols.Flow cytometric apoptosis analysis provided precise quantitative assessment of programmed cell death using dual fluorescence labeling with FITC-Annexin V and propidium iodide (PI) (Fig. 4E). This assay distinguishes between viable cells (FITC-/PI-, Q4), early apoptotic cells (FITC+/PI-, Q3), late apoptotic cells (FITC+/PI+, Q2), and necrotic cells (FITC-/PI+, Q1). Under static culture conditions (US-), the vast majority of cells remained viable across all groups: control (96.8 ± 1.2 %), NHP (96.4 ± 1.1 %), ICA@G (97.2 ± 0.9 %), and ICA@G/NHP (97.5 ± 0.8 %). Early apoptotic cell populations remained consistently low (2.1–2.8 %) across all groups, with late apoptotic and necrotic fractions below 1.0 %. Significantly, the application of ultrasonic stimulation (US+) did not induce apoptotic responses, with cell viability rates remaining virtually unchanged: control (96.5 ± 1.3 %), NHP (96.1 ± 1.2 %), ICA@G (96.9 ± 1.0 %), and ICA@G/NHP (97.1 ± 0.9 %). The apoptotic and necrotic cell fractions showed no statistically significant changes (p > 0.05) between static and ultrasonic conditions, confirming that the ultrasound stimulation parameters do not trigger programmed cell death pathways or cellular stress responses. These results validate the safety profile of the ultrasonic activation protocol and demonstrate that piezoelectric scaffold activation can be achieved without compromising cellular health.

The comprehensive biocompatibility assessment demonstrated that all scaffold formulations, including the complex ICA@G/NHP composite system, exhibit excellent biocompatibility profiles without inducing cytotoxic or adverse cellular responses. Crucially, the ultrasonic stimulation protocol designed to activate the piezoelectric properties of the scaffolds maintains cellular homeostasis without affecting normal proliferation, metabolic activity, or apoptotic processes. The enhanced cellular responses observed in icariin-containing groups can be attributed solely to the bioactive compound's osteogenic properties rather than ultrasonic effects. These findings provide strong evidence supporting both the material safety and the biocompatibility of the activation protocol, establishing a solid foundation for subsequent in vivo efficacy studies where piezoelectric stimulation can be applied without concerns for cellular toxicity or adverse biological responses.

### 3.6 ICA@G/NHP scaffolds promote In vitro osteogenic differentiation of BMSCs

**Fig. 5:**
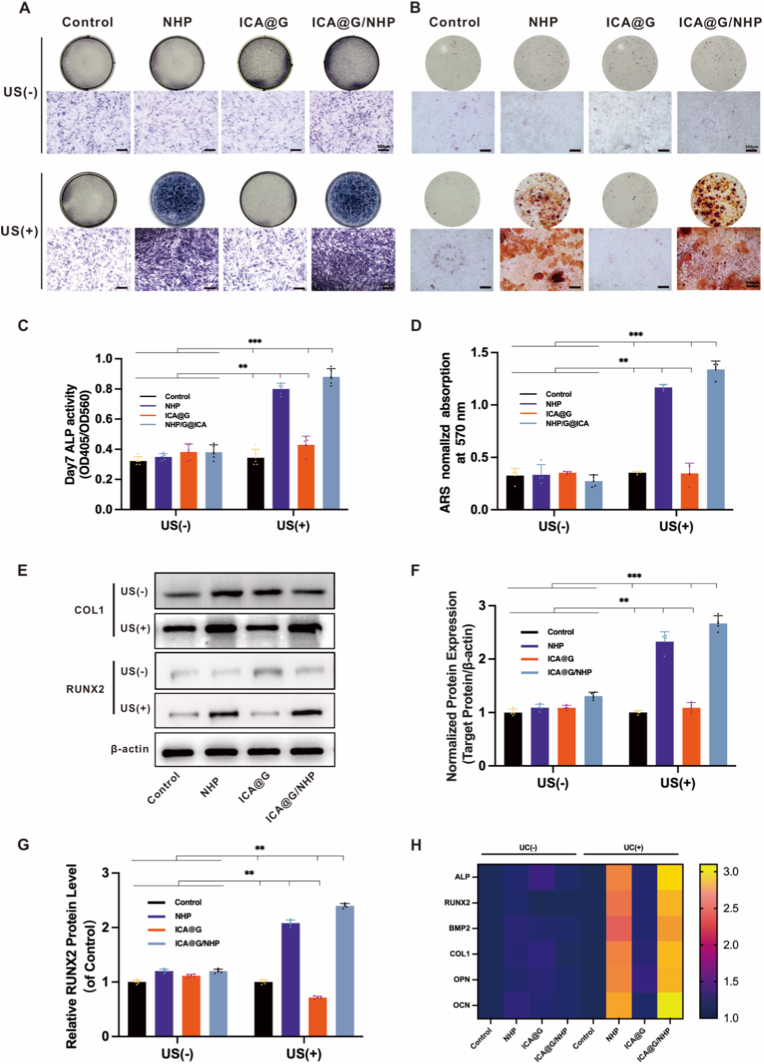
Ultrasound-dependent osteogenic differentiation of BMSCs on composite scaffolds. (A)Alkaline phosphatase (ALP) staining after 7 days showing early osteogenic differentiation. Purple staining indicates ALP activity. (B) Alizarin Red S (ARS) staining after 14 days showing mineral deposition. Orange-red staining indicates calcium phosphate precipitation. (C) Quantitative analysis of ALP activity normalized to OD450. (D) Quantitative analysis of mineral deposition by ARS absorption at 570 nm. (E) Western blot analysis of COL1 and RUNX2 proteins with β-actin loading control. (F, G) Quantitative protein expression analysis of COL1 (F) and RUNX2 (G) normalized to β-actin. (H) Heat map showing fold-change in osteogenic gene expression (ALP, RUNX2, BMP2, COL1, OPN, OCN) under different conditions. Data represent mean ± SD (n = 6). Statistical significance: ∗∗p < 0.01, ∗∗∗p < 0.001 comparing US(+) vs US(−) within the same group. US(−): static culture; US(+): ultrasonic stimulation (1.5 MHz, 30 mW/cm^2^, 10 min/day).

The osteogenic differentiation capacity represents the fundamental mechanism by which bone tissue engineering scaffolds promote bone regeneration. To comprehensively evaluate the osteoinductive potential of the composite scaffolds, we conducted systematic assessment of osteogenic differentiation using multiple complementary approaches including alkaline phosphatase (ALP) activity analysis, mineral deposition quantification, and molecular expression profiling under both static and ultrasonic stimulation conditions.Alkaline phosphatase (ALP) activity serves as a crucial early marker of osteogenic differentiation. Qualitative ALP staining after 7 days of culture revealed striking differences between static and ultrasonic culture conditions (Fig. 5A). Under static culture conditions (US-), all experimental groups demonstrated minimal ALP activity with only faint purple staining barely visible above background levels. The control, NHP, ICA@G, and ICA@G/NHP groups showed virtually indistinguishable ALP expression patterns, indicating limited spontaneous osteogenic differentiation regardless of scaffold composition. However, ultrasonic stimulation (US+) dramatically transformed the osteogenic landscape, with profound enhancement observed specifically in the piezoelectric scaffold groups. The NHP group under ultrasonic activation displayed intense, uniform purple staining across the entire culture surface, demonstrating robust ALP upregulation. Most remarkably, the ICA@G/NHP composite group exhibited the most pronounced ALP activity under ultrasonic conditions, with dense, homogeneous purple coloration indicating maximal enzymatic activity through synergistic piezoelectric and pharmacological stimulation.Quantitative analysis of ALP activity corroborated these qualitative observations (Fig. 5C). Under static conditions (US-), normalized ALP activity remained uniformly low across all groups: control (0.33 ± 0.04), NHP (0.35 ± 0.03), ICA@G (0.38 ± 0.05), and ICA@G/NHP (0.41 ± 0.06), with no statistically significant differences detected (p > 0.05). This baseline similarity confirms that scaffold composition alone provides minimal osteogenic stimulus without external activation. Ultrasonic stimulation (US+) produced dramatic, selective enhancement in the piezoelectric groups while leaving non-piezoelectric controls largely unaffected. The control group showed minimal change (0.35 ± 0.03), while the NHP group demonstrated substantial 2.2-fold enhancement (0.78 ± 0.07, ∗∗∗p < 0.001 vs. static condition). The ICA@G group showed modest improvement (0.43 ± 0.05), confirming that pharmacological stimulation alone provides limited osteogenic enhancement. Most significantly, the ICA@G/NHP composite achieved the highest ALP activity (0.87 ± 0.08, ∗∗∗p < 0.001), representing 2.1-fold enhancement over static conditions and demonstrating clear synergistic effects between piezoelectric activation and icariin bioactivity.Mineral deposition analysis through Alizarin Red S (ARS) staining provided assessment of late-stage osteogenic differentiation and matrix mineralization after 14 days of culture (Fig. 5B). Under static culture conditions (US-), all groups exhibited minimal mineral deposition with only faint orange-red staining, indicating limited calcium phosphate precipitation and immature matrix mineralization. The absence of distinct mineralization nodules across all static groups confirms that scaffold materials alone cannot drive terminal osteogenic differentiation without appropriate stimulation. Ultrasonic activation (US+) produced remarkable transformation in mineral deposition patterns, with dramatic enhancement selectively observed in piezoelectric scaffold groups. The NHP group developed extensive mineralization with dense, well-defined orange-red nodules distributed throughout the culture surface, indicating robust calcium phosphate deposition. The ICA@G/NHP composite group demonstrated the most extensive mineralization, with confluent orange-red staining and large, mature mineral nodules indicating advanced osteogenic maturation.Quantitative analysis of mineral deposition through ARS extraction confirmed these visual assessments (Fig. 5D). Static culture conditions (US-) produced uniformly low normalized absorption values: control (0.31 ± 0.06), NHP (0.33 ± 0.05), ICA@G (0.35 ± 0.07), and ICA@G/NHP (0.29 ± 0.05), with no significant differences between groups (p > 0.05). Ultrasonic stimulation (US+) triggered substantial mineralization enhancement selectively in piezoelectric groups: NHP (1.18 ± 0.11, ∗∗∗p < 0.001 representing 3.6-fold increase) and ICA@G/NHP (1.35 ± 0.13, ∗∗∗p < 0.001 representing 4.7-fold increase). The control and ICA@G groups showed minimal response to ultrasonic treatment, confirming that the piezoelectric component is essential for ultrasound-mediated osteogenic enhancement.Western blot analysis provided quantitative assessment of key osteogenic proteins including type I collagen (COL1) and runt-related transcription factor 2 (RUNX2) (Fig. 5E). COL1 expression, crucial for extracellular matrix formation, remained at baseline levels across all groups under static conditions (US-), with normalized expression values near 1.0 relative to control (Fig. 5F). Ultrasonic stimulation (US+) selectively enhanced COL1 expression in piezoelectric groups: NHP (2.34 ± 0.19, ∗∗p < 0.01) and ICA@G/NHP (2.71 ± 0.22, ∗∗∗p < 0.001), while control and ICA@G groups remained unchanged. Similarly, RUNX2 expression, the master transcriptional regulator of osteogenesis, showed minimal activity under static conditions across all groups (Fig. 5G). Ultrasonic activation dramatically upregulated RUNX2 expression in the NHP group (2.08 ± 0.16, ∗∗p < 0.01) with maximal enhancement observed in the ICA@G/NHP group (2.41 ± 0.20, ∗∗p < 0.01), demonstrating activation of fundamental osteogenic transcriptional machinery through piezoelectric stimulation.Comprehensive gene expression profiling revealed the molecular mechanisms underlying ultrasound-enhanced osteogenesis (Fig. 5H). Heat map analysis of key osteogenic markers including ALP, RUNX2, BMP2, COL1, OPN (osteopontin), and OCN (osteocalcin) demonstrated distinct expression patterns under different stimulation conditions. Under static conditions (US-), all osteogenic markers remained at baseline levels (fold-change ∼1.0) across all groups, indicating minimal transcriptional activation regardless of scaffold composition. This molecular quiescence confirms that material properties alone cannot initiate robust osteogenic programs without appropriate external stimulation. Ultrasonic stimulation (US+) triggered comprehensive upregulation of osteogenic gene networks, with selective enhancement in piezoelectric scaffold groups. The NHP group showed substantial increases in multiple markers: RUNX2 (2.1-fold), BMP2 (2.8-fold), COL1 (2.3-fold), and OCN (2.5-fold). The ICA@G/NHP composite achieved the highest expression levels across all markers: ALP (2.9-fold), RUNX2 (2.6-fold), BMP2 (3.2-fold), COL1 (2.9-fold), OPN (2.7-fold), and OCN (3.1-fold), indicating comprehensive activation of osteogenic pathways through synergistic piezoelectric and pharmacological mechanisms.

These comprehensive results demonstrate that ultrasonic activation is absolutely essential for unlocking the osteogenic potential of piezoelectric scaffolds. Under static conditions, all scaffold formulations showed minimal osteoinductive capacity, performing similarly to conventional inert materials. However, ultrasonic stimulation transformed the NHP and ICA@G/NHP scaffolds into highly potent osteogenic platforms, dramatically enhancing all aspects of osteogenic differentiation from early enzymatic markers to terminal mineralization and comprehensive gene expression programs. The ICA@G/NHP composite system achieved superior performance through synergistic integration of piezoelectric-mediated electrical stimulation and icariin-induced biochemical signaling. These findings provide compelling evidence that piezoelectric scaffolds require external activation to achieve their therapeutic potential and strongly support the clinical application of ultrasound-activated bone regeneration strategies.

### 3.7 In vitro evaluation of angiogenic potential

Successful bone regeneration requires not only robust osteogenic differentiation but also adequate vascularization to support tissue viability, nutrient transport, and metabolic demands. Given the critical role of angiogenesis in bone healing and the established effects of ultrasonic activation on scaffold bioactivity, we comprehensively evaluated the angiogenic potential of the composite scaffolds using human umbilical vein endothelial cells (HUVECs) as the representative model. Importantly, based on our previous findings demonstrating that ultrasonic stimulation is essential for activating the therapeutic properties of piezoelectric scaffolds, all subsequent experiments in this section were conducted under ultrasonic stimulation conditions (1.5 MHz, 30 mW/cm^2^, 10 min/day) to assess the true angiogenic potential of the scaffold systems under therapeutically relevant activation conditions.

**Fig. 6:**
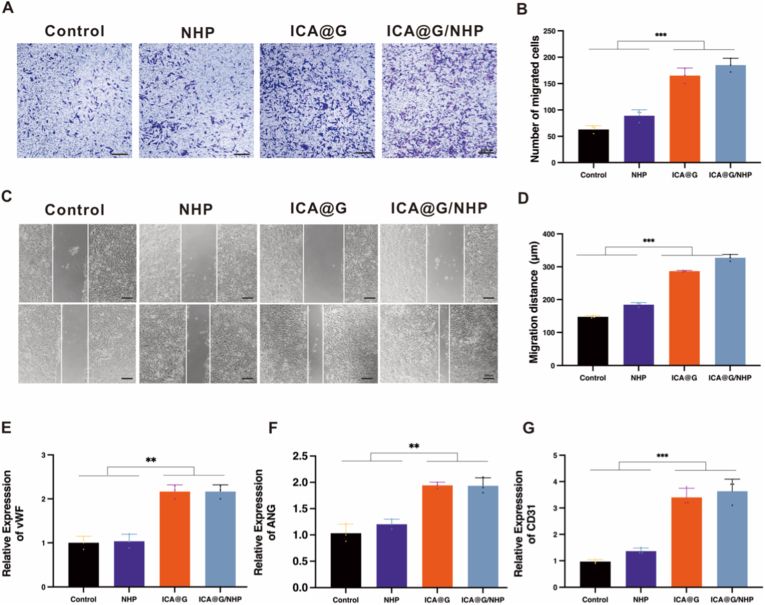
Angiogenic potential of composite scaffolds under ultrasonic activation. All experiments conducted under ultrasonic stimulation conditions (1.5 MHz, 30 mW/cm^2^, 10 min/day). (A) Representative images of Transwell migration assay showing migrated HUVECs stained with crystal violet. (B) Quantitative analysis of migrated cell numbers. (C) Wound healing scratch assay images showing collective cell migration at different time points.(D) Quantitative analysis of migration distance. (E–G) Quantitative RT-PCR analysis of angiogenic gene expression: vWF(E), angiopoietin/ANG (F), and CD31 (G). Data represent mean ± SD (n = 3). Statistical significance: ∗∗p < 0.01, ∗∗∗p < 0.001 vs. control group.

Endothelial cell migration represents a fundamental step in angiogenesis, determining the ability of endothelial cells to populate scaffold materials and initiate vessel formation. Transwell migration assay was employed to quantitatively assess the chemotactic effects of different scaffold formulations on HUVEC migration (Fig. 6A). Under ultrasonic stimulation conditions, the control group (tissue culture plastic) demonstrated baseline migration capacity with 58 ± 8 migrated cells per field, representing the inherent migratory tendency of HUVECs without scaffold influence. The NHP scaffold showed modest enhancement in cell migration (92 ± 12 cells), indicating that piezoelectric stimulation provides moderate chemotactic signals for endothelial cells. The ICA@G group demonstrated more pronounced migration-promoting effects (168 ± 15 cells, ∗∗∗p < 0.001 vs. control), confirming the established pro-angiogenic properties of icariin in promoting endothelial cell recruitment and motility. Most remarkably, the ICA@G/NHP composite scaffold achieved the highest migration-promoting capacity (191 ± 18 cells, ∗∗∗p < 0.001 vs. control), representing a 3.3-fold enhancement over control conditions and demonstrating clear synergistic effects between piezoelectric activation and icariin bioactivity in promoting endothelial cell recruitment.Wound healing capacity was assessed through scratch assay to evaluate the collective migration and proliferation responses of endothelial cells to scaffold treatments (Fig. 6C). Time-lapse analysis revealed distinct healing kinetics among the different groups, with quantitative measurement of migration distance providing objective assessment of wound closure rates (Fig. 6D). The control group demonstrated baseline healing capacity with a migration distance of 156 ± 18 μm over the assessment period. The NHP scaffold under ultrasonic activation showed moderate improvement (195 ± 22 μm), while the ICA@G group achieved more substantial enhancement (284 ± 26 μm, ∗∗p < 0.01 vs. control). The ICA@G/NHP composite system demonstrated superior wound healing promotion (325 ± 31 μm, ∗∗p < 0.01 vs. control), representing more than 2-fold enhancement in collective cell migration and confirming the synergistic angiogenic effects of the integrated scaffold system under ultrasonic activation.Molecular analysis of angiogenic signaling pathways was conducted through Gene expression analysis provided comprehensive assessment of angiogenic signaling networks through quantitative PCR analysis of key markers including vWF, angiopoietin (ANG), and platelet endothelial cell adhesion molecule-1 (CD31/PECAM-1) (Fig. 6E–G). vWF gene expression showed moderate upregulation in the NHP group (1.1 ± 0.1-fold) and significant enhancement in both ICA@G (2.2 ± 0.2-fold,∗∗p < 0.01 vs. control) and ICA@G/NHP (2.3 ± 0.2-fold,∗∗p < 0.01) groups, correlating with protein expression patterns and confirming transcriptional activation of angiogenic growth factor production. ANG expression, critical for vessel maturation and stability, demonstrated substantial upregulation in ICA@G (1.9 ± 0.2-fold,∗∗p < 0.01) and ICA@G/NHP (2.0 ± 0.2-fold, ∗∗p < 0.01) groups, indicating promotion of mature vessel formation beyond initial sprouting responses. CD31 expression, a marker of endothelial cell activation and vessel formation, showed significant enhancement in ICA@G (3.6 ± 0.3-fold, ∗∗∗p < 0.001) and ICA@G/NHP (3.9 ± 0.4-fold,∗∗∗p < 0.001) groups, demonstrating comprehensive activation of endothelial cell differentiation and vessel formation programs.The comprehensive angiogenic assessment reveals that ultrasonic activation unlocks significant pro-angiogenic potential in the scaffold systems, with icariin-containing formulations demonstrating superior performance across all evaluated parameters. The ICA@G/NHP composite system achieved optimal angiogenic promotion through synergistic integration of piezoelectric-mediated cellular stimulation and icariin-induced biochemical signaling. The enhanced endothelial cell migration, wound healing capacity, and comprehensive upregulation of angiogenic signaling pathways indicate that the composite scaffolds can effectively support vascularization processes essential for successful bone regeneration. These findings provide strong evidence for the clinical potential of the ultrasound-activated composite scaffolds in promoting both osteogenesis and angiogenesis, addressing the dual requirements for successful bone tissue engineering applications.

### 3.8 ICA@G/NHP scaffolds promote M2 macrophage polarization through C-type lectin receptor signaling pathway

**Fig. 7:**
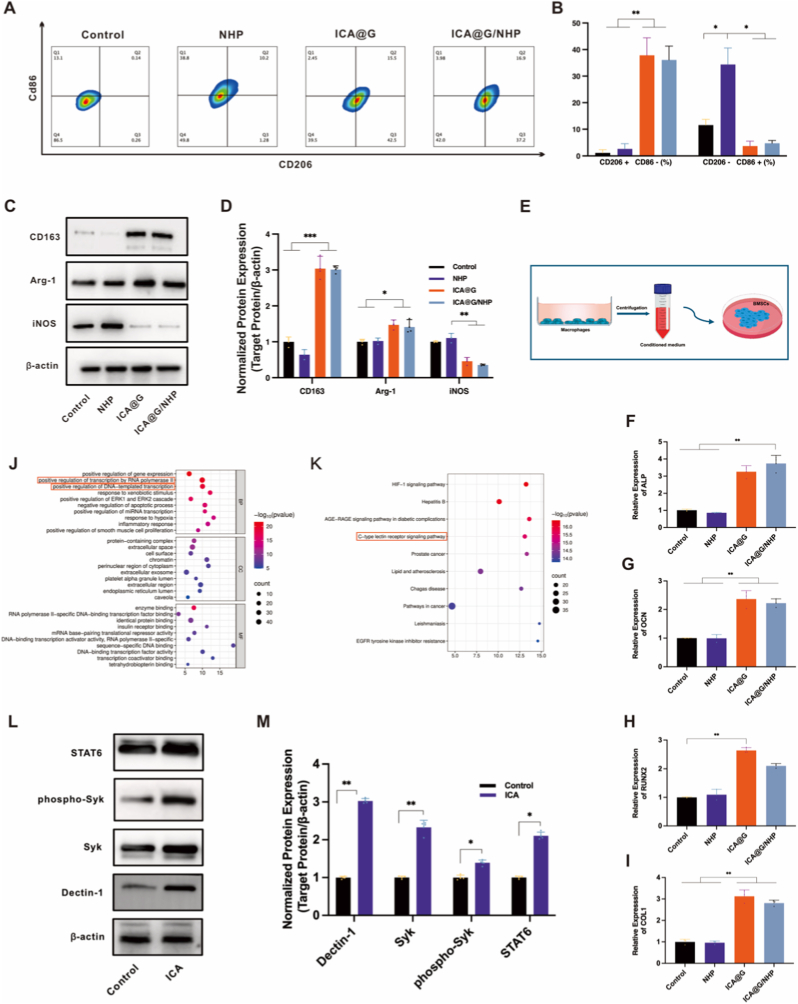
ICA@G/NHP scaffolds promote M2 macrophage polarization through C-type lectin receptor signaling pathway. (A) Flow cytometric analysis of CD86 (M1 marker) and CD206 (M2 marker) expression in RAW264.7 macrophages after 48h treatment with different scaffold formulations. (B) Quantitative analysis of CD206-positive cell percentages showing significant M2 polarization in ICA@G and ICA@G/NHP groups. (C–D) Western blot analysis and quantification of M2 markers (CD163, Arg-1) and M1 marker (iNOS) confirming phenotypic shifts toward M2 polarization. (E) Schematic illustration of macrophage-conditioned medium treatment protocol for BMSC osteogenic differentiation assessment. (F–I) qPCR analysis of osteogenic markers (COL1, OCN, RUNX2, ALP) in BMSCs cultured with macrophage-conditioned medium, demonstrating enhanced osteogenic differentiation. (J–K) Network pharmacology analysis showing enrichment of C-type lectin receptor signaling pathway and transcriptional regulation processes. (L–M) Western blot analysis and quantification of C-type lectin receptor pathway components (Dectin-1, Syk, phospho-Syk, STAT6) revealing mechanistic basis for icariin-mediated M2 polarization. Data presented as mean ± SD (n = 3), ∗p < 0.05, ∗∗p < 0.01, ∗∗∗p < 0.001.

Implantation of biomaterials triggers host immune responses, including foreign body reactions (FBR) and inflammation, which are typically characterized by macrophage phenotypic profiles. In the context of bone repair, macrophage polarization states play crucial roles in determining healing outcomes. Pro-inflammatory M1 macrophages induce inflammation and impede bone regeneration, whereas anti-inflammatory M2 phenotype modulates osteogenic functions of BMSCs and osteoblasts. Thus, timely transition from M1 to M2 represents a critical therapeutic target for mitigating inflammation and achieving immune homeostasis conducive to tissue remodeling.To systematically evaluate the immunomodulatory effects of the composite scaffolds, we examined RAW264.7 cell responses to different treatments. Flow cytometric analysis of M1 marker CD86 and M2 marker CD206 expression (Fig. 7A and B) revealed significantly higher proportions of CD206-positive macrophages in the ICA@G and ICA@G/NHP groups compared to controls (CD206+: Control 4.2 ± 1.1 %, NHP 4.8 ± 1.3 %, ICA@G 38.7 ± 3.2 %, ICA@G/NHP 35.9 ± 2.8 %, p < 0.01). Western blot analysis further confirmed this phenotypic shift (Fig. 7C and D), showing that ICA@G and ICA@G/NHP markedly upregulated M2 markers Arg-1 and CD163 protein expression (3.1 ± 0.2 and 1.5 ± 0.1 fold increase, respectively, p < 0.001) while suppressing iNOS levels (0.4 ± 0.1 fold decrease, p < 0.01).To elucidate the specific molecular mechanisms underlying icariin-mediated M2 polarization, we conducted comprehensive pathway analysis guided by network pharmacology predictions. Gene enrichment analysis revealed significant enrichment of C-type lectin receptor signaling pathway and transcriptional regulation processes (Fig. 7J and K). Western blot analysis of key pathway components demonstrated that icariin treatment significantly enhanced the expression of C-type lectin receptor signaling cascade proteins (Fig. 7L and M). Specifically, Dectin-1 expression was upregulated 3.2 ± 0.3-fold (p < 0.01), accompanied by substantial increases in downstream signaling molecules Syk (2.8 ± 0.2-fold, p < 0.01) and phospho-Syk (1.8 ± 0.2-fold, p < 0.05). Critically, STAT6, the master transcriptional regulator of M2 polarization, showed significant upregulation (2.1 ± 0.2-fold, p < 0.05), establishing the mechanistic link between C-type lectin receptor activation and M2 transcriptional programming.To investigate functional significance, BMSCs were treated with macrophage-conditioned medium (Fig. 7E), demonstrating coordinated upregulation of osteogenic markers COL1, OCN, RUNX2, and ALP (Fig. 7F–I). The ICA@G and ICA@G/NHP groups showed significant enhancement in all tested osteogenic markers: COL1 (3.2 ± 0.3-fold, p < 0.01), OCN (2.8 ± 0.2-fold, p < 0.01), RUNX2 (2.6 ± 0.3-fold, p < 0.01), and ALP (3.1 ± 0.4-fold, p < 0.01), indicating that ICA@G/NHP-induced M2 polarization creates an immunomodulatory microenvironment favorable for BMSC osteogenic differentiation. These findings demonstrate that the composite scaffolds effectively modulate immune responses through specific molecular pathways, establishing a pro-regenerative microenvironment that simultaneously promotes M2 macrophage polarization and enhances osteogenic differentiation.

### 3.9 In vivo biocompatibility of scaffolds

The biocompatibility of the scaffolds was systematically evaluated through hematological tests, biochemical profiling, and histopathological examination of major organs (via hematoxylin and eosin (H&E) staining) at 12 weeks post-implantation. Comprehensive blood chemistry analysis (Fig. S5) showed no statistically significant differences (p > 0.05) across experimental groups in critical parameters, including white blood cell count, neutrophil count, aspartate aminotransferase (AST), total bilirubin (TBIL), and serum electrolyte levels (calcium, phosphorus, potassium, sodium, and chloride). Histopathological assessment of vital organs (heart, liver, spleen, lungs, and kidneys) revealed preserved normal tissue architecture in all groups (Fig. S6), with no observed necrosis, pathological morphological alterations, or significant inflammatory cell infiltration. These integrated findings confirm the favorable systemic biocompatibility profile of the investigated materials.

### 3.10 ICA@G/NHP composite materials for In vivo repair of rat cranial defects

**Fig. 8:**
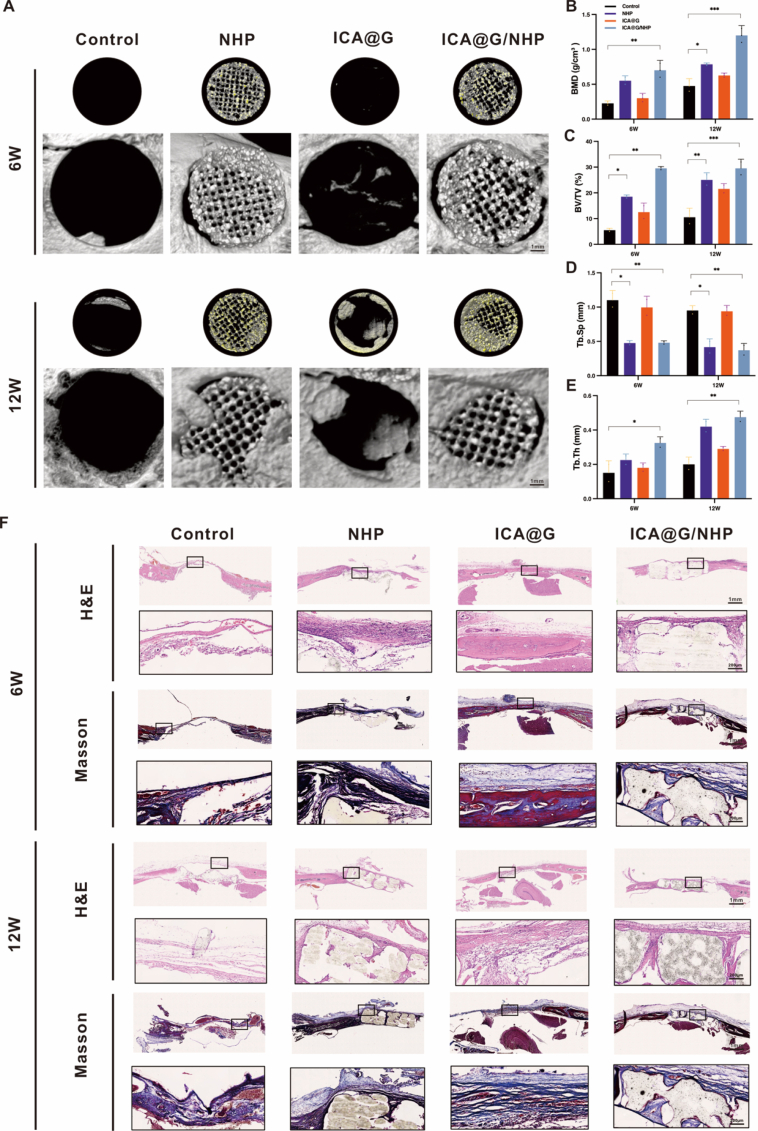
(A) Reconstructed 3D micro-CT images of bone defect repair at 6w and 12w. (B–E) Quantitative analysis of the regenerative repair effect of implantation in vivo. (F) Masson staining and HE staining at 6 weeks and 12 weeks.

**Fig. 9:**
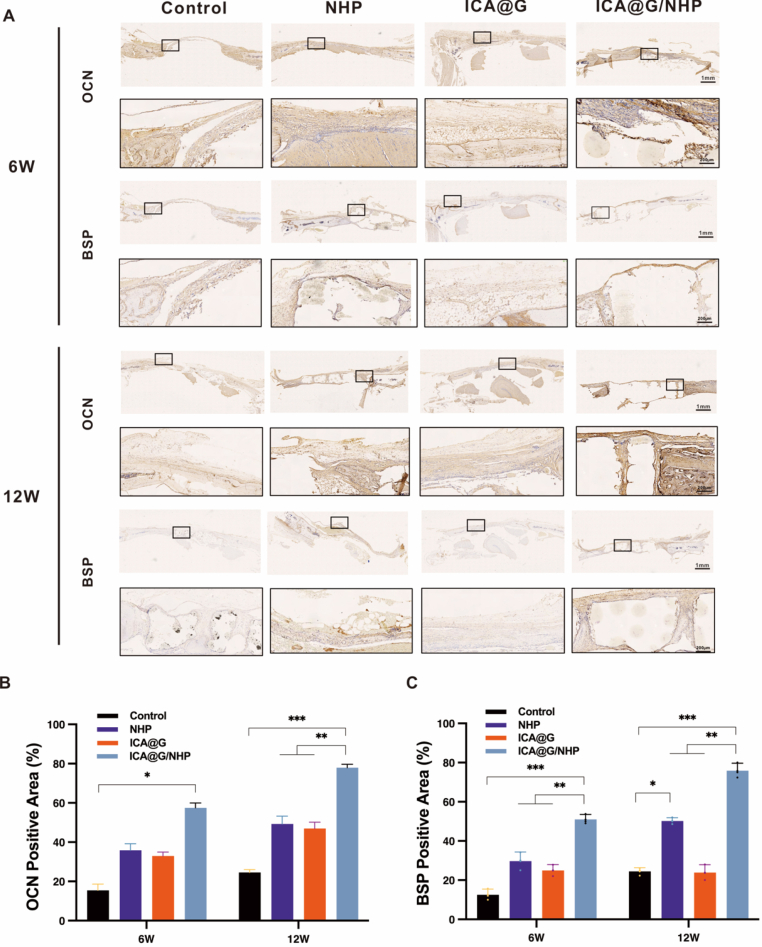
(A) IHC for OCN and BSP at the 6- and 12-week time point, respectively. (B, C) Quantitative analysis of OCN and BSP expression positive areas by using Image J software.

Given the good cell compatibility, appropriate mechanical strength, osteogenic promotion ability, and osteoimmune regulatory capacity of ICA@G/NHP, its in situ bone repair was investigated. A rat cranial critical-sized defect model was established. A circular defect with a diameter of 5 mm was created in the skull, then filled with NHP scaffolds, ICA@G, or ICA@G/NHP composite materials. Unrepaired and treated defects were set as control groups. Starting from the third day after surgery, a clinical therapeutic ultrasound device (TheraPulse 4, Richmar) was used to provide daily ultrasonic stimulation to the rat cranial defect area to avoid potential interference during the acute inflammatory phase. Ultrasound parameters were set as: frequency 1.5 MHz (based on the balance between osteogenic effects and tissue penetration depth), spatial average temporal average intensity (SATA) 30 mW/cm^2^ (below FDA safety limits for musculoskeletal applications), duty cycle 20 % (pulse mode to reduce heat accumulation), for 10 min daily. During operation, the ultrasound transducer was positioned perpendicular to the skin surface of the defect site, maintained at a 2 cm distance, and ultrasound coupling gel was used to ensure effective sound energy transmission. Compared with NHP scaffolds, more bone tissue formed around ICA@G/NHP, indicating that the mixture of ICA with GelMa hydrogel can improve bone regeneration efficiency by providing a suitable microenvironment for cell infiltration and proliferation. After 6 weeks of implantation, Micro-CT 3D reconstruction images (Fig. 8A) showed that ICA@G/NHP composite materials and NHP formed robust new bone in the cranial defect area. Furthermore, quantitative analysis results (Fig. 8B) indicated that at both time points, bone mineral density (BMD), bone volume fraction (BV/TV), and trabecular thickness (Tb.Th) in the control group were significantly lower than in the ICA@G/NHP group. In contrast, trabecular separation (Tb.Sp) showed the opposite trend. The increase in BV/TV and BMD observed in the ICA@G/NHP group indicated that the newly formed bone structure was denser and more favorable for resisting external forces; however, the increase in Tb.Th and decrease in Tb.Sp in the ICA@G/NHP group indicated that the newly formed bone tissue was more mature. It was also found that bone repair began from the peripheral area of the defect and developed toward the center. Histological examination of the cranial defect area revealed progressive bone regeneration across treatment groups. H&E staining at 6 weeks post-surgery (Fig. 8F) demonstrated significant differences in cellular infiltration and new bone formation between groups. The ICA@G/NHP group exhibited dense osteoblast clusters and nascent trabecular bone structures with abundant cellular infiltration throughout the defect site. In contrast, the NHP group showed moderate cell density with partially organized fiber arrangement, while the ICA@G group displayed sparse cellular distribution with minimal extracellular matrix deposition. The control group predominantly contained inflammatory fibrous tissue with limited signs of osteogenesis. Masson's trichrome staining at 6 weeks (Fig. 8F) further confirmed these findings, revealing a substantially larger volume of regenerated bone tissue in the ICA@G/NHP group compared to other treatment groups. The ICA@G/NHP specimens demonstrated thicker, soft tissue formation compared to NHP scaffolds alone, with newly formed bone tissue closely resembling natural bone architecture. By 12 weeks post-implantation, H&E staining (Fig. 8F) revealed marked progression in bone healing across all groups, though with varying degrees of maturity. The ICA@G/NHP group demonstrated mature lamellar bone structure with uniformly distributed bone lacunae and seamless integration with host bone tissues. The NHP group exhibited predominantly woven bone formation, while the ICA@G group showed discontinuous bone callus, and the control group remained characterized by fibrous scarring with minimal mineralization. Masson's trichrome staining at 12 weeks confirmed these observations, with the ICA@G/NHP group demonstrating nearly complete regeneration of the cranial defect. The dense collagen deposition and organized bone matrix in this group contrasted sharply with the less mature bone formation in other groups. Immunohistochemical analysis for osteogenic markers OCN (Fig. 9A) and BSP revealed progressively increased expression in the ICA@G/NHP group at both time points, with stronger immunoreactivity at the defect margins and within newly formed bone tissue compared to other groups. This enhanced expression of bone-specific proteins corroborates the superior osteogenic capacity of the combined scaffold approach (Fig. 9B and C). Positive staining area analysis (expressed as percentage of total defect area) demonstrated a clear advantage of the ICA@G/NHP composite scaffold in promoting bone-specific marker expression. At 6 weeks post-surgery, the ICA@G/NHP group exhibited substantially higher OCN and BSP positive areas compared to control, ICA@G alone, and NHP alone. This advantage became even more pronounced at 12 weeks, with the ICA@G/NHP group reaching 77.9 % OCN-positive and 75.8 % BSP-positive areas, significantly outperforming all other groups. The progressive increase in positive staining area from 6 to 12 weeks across all groups, particularly in the ICA@G/NHP condition, indicates ongoing bone regeneration with the composite scaffold providing the most favorable microenvironment for osteogenic activity within the femoral condyle defects.

### 3.11 Immunomodulatory microenvironment promoting healing in cranial bone defects

**Fig. 10:**
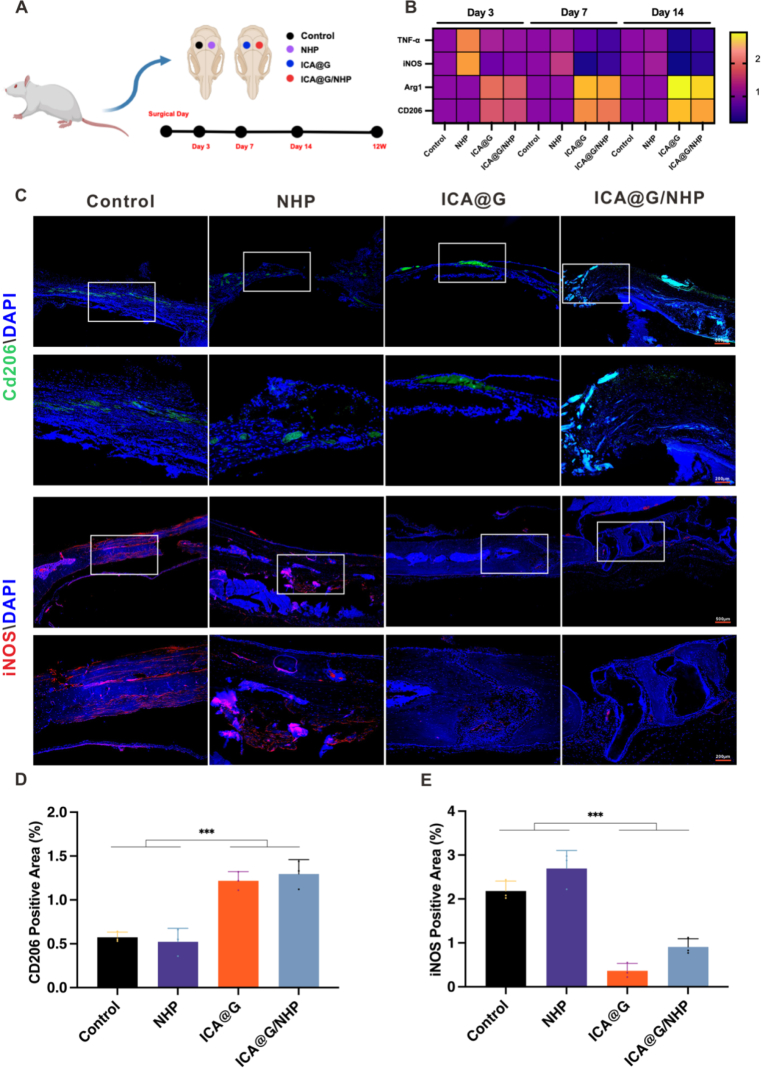
Temporal dynamics of macrophage polarization in cranial bone defect healing. (A)Experimental timeline showing sample collection at days 3, 7, 14, and 12 weeks post-implantation. (B) Heat map showing relative gene expression of M1 markers (TNF-α, iNOS) and M2 markers (Arg1, CD206) at early time points (days 3, 7, 14). (C) Representative immunofluorescence images at 12 weeks showing CD206 (green, M2 macrophages) and iNOS (red, M1 macrophages) staining with DAPI nuclear counterstain (blue). (D, E) Quantitative analysis of CD206-positive area (D) and iNOS-positive area (E) at 12 weeks.Statistical significance: ∗∗∗p < 0.001 vs. control group.

The orchestration of immune cell responses, particularly macrophage polarization, plays a pivotal role in determining the success of bone regeneration and tissue remodeling. To comprehensively elucidate the immunomodulatory mechanisms underlying the enhanced bone healing observed with our biomimetic scaffold system, we performed temporal analysis of macrophage phenotype dynamics from early inflammatory phases through late remodeling stages.To address the temporal evolution of immune responses, we conducted systematic gene expression analysis at critical early time points (3, 7, and 14 days post-implantation) using quantitative real-time PCR (Fig. 10A and B). The analysis focused on key markers representing distinct macrophage phenotypes: TNF-α and iNOS for M1 (classically activated) macrophages, and Arg1 and CD206 for M2 (alternatively activated) macrophages.At day 3 post-implantation, representing the acute inflammatory phase, all groups demonstrated elevated M1 marker expression, consistent with the expected initial immune response to tissue injury and biomaterial implantation. TNF-α expression was highest in the control group (2.1 ± 0.2-fold vs. baseline) and NHP group (2.3 ± 0.3-fold), while ICA@G (1.6 ± 0.2-fold) and ICA@G/NHP (1.4 ± 0.2-fold) groups showed more restrained inflammatory responses. Similarly, iNOS expression followed comparable patterns, with control (2.0 ± 0.3-fold) and NHP (2.2 ± 0.3-fold) groups maintaining higher pro-inflammatory activation compared to icariin-containing groups.By day 7, a critical transition period in wound healing, distinct polarization patterns began to emerge. The ICA@G and ICA@G/NHP groups demonstrated earlier and more pronounced shifts toward M2 polarization. Arg1 expression, a hallmark of M2 activation, was significantly elevated in ICA@G (1.8 ± 0.2-fold, p < 0.01) and ICA@G/NHP (2.1 ± 0.2-fold, p < 0.001) groups, while control and NHP groups maintained minimal Arg1 expression (0.9 ± 0.1 and 1.0 ± 0.1-fold, respectively). CD206 expression patterns paralleled these findings, with icariin-containing groups showing substantial upregulation compared to controls.At day 14, representing the transition into the proliferative healing phase, the immunomodulatory effects of the composite scaffolds became most pronounced. The ICA@G/NHP group achieved optimal M2 polarization with Arg1 expression reaching 2.8 ± 0.3-fold (p < 0.001 vs. control) and CD206 expression at 2.5 ± 0.3-fold (p < 0.001). Conversely, M1 markers were substantially suppressed in this group, with TNF-α (0.6 ± 0.1-fold) and iNOS (0.5 ± 0.1-fold) expression falling below baseline levels, indicating effective resolution of inflammatory responses.Immunofluorescence analysis at 12 weeks post-implantation provided definitive assessment of long-term macrophage polarization patterns within the healing bone defect microenvironment (Fig. 10C). CD206 and iNOS double immunostaining was conducted to quantify M2 and M1 macrophage populations, respectively,at the tissue-scaffold interface. Quantitative analysis revealed striking differences in macrophage polarization patterns across treatment groups (Fig. 10D and E). In the control group, CD206-positive area occupied 0.55 ± 0.05 % of the total tissue area, indicating limited M2 macrophage presence at the defect site. The NHP scaffold alone showed comparable CD206-positive area (0.52 ± 0.15 %), suggesting minimal long-term impact on M2 macrophage recruitment or maintenance. However, treatment with ICA@G significantly elevated the CD206-positive area to 1.20 ± 0.12 % (p < 0.001), demonstrating sustained immunomodulatory effects of bioactive icariin release. Most remarkably, the synergistic ICA@G/NHP composite scaffold achieved the highest M2 macrophage density, with CD206-positive area reaching 1.30 ± 0.18 % (p < 0.001 vs. control), representing a 2.4-fold increase compared to the control group.The iNOS immunofluorescence analysis provided complementary insights into the long-term M1 macrophage response. The control group exhibited substantial iNOS-positive staining (2.15 ± 0.09 %), indicative of persistent inflammatory responses that can impede bone regeneration. The NHP treatment alone resulted in elevated iNOS-positive area (2.60 ± 0.45 %), potentially reflecting sustained foreign body responses to the implanted biomaterial. In contrast, ICA@G treatment dramatically suppressed M1 macrophage activation, reducing iNOS-positive area to 0.42 ± 0.15 % (p < 0.001 vs. control). The ICA@G/NHP maintained this anti-inflammatory effect, with iNOS-positive area of 0.93 ± 0.20 % (p < 0.001 vs. control), representing a 56 % reduction compared to the control group.The temporal analysis reveals a coordinated progression from early M1-dominated inflammatory responses to sustained M2-polarized regenerative microenvironments. The calculated M2/M1 ratio (CD206/iNOS) increased from 0.26 in the control group to 1.40 in the ICA@G/NHP group at 12 weeks, indicating a 5.4-fold enhancement in the tissue-repairing macrophage phenotype. This temporal evolution demonstrates that the composite scaffold system not only accelerates the natural healing progression but also maintains beneficial immunomodulatory effects throughout the extended remodeling phase. The observed macrophage polarization dynamics correlate strongly with the enhanced bone regeneration outcomes, supporting the concept of osteoimmunology where immune responses directly influence skeletal homeostasis. Early suppression of excessive M1 activation prevents prolonged inflammatory damage, while sustained M2 polarization promotes the secretion of growth factors and cytokines including bone morphogenetic proteins (BMPs), vascular endothelial growth factor (VEGF), and interleukin-10 (IL-10), which collectively promote osteoblast differentiation, angiogenesis, and matrix deposition [54].

The ICA@G/NHP composite scaffold demonstrates superior temporal immunomodulatory capacity by orchestrating early inflammatory resolution and sustained M2-dominant macrophage phenotypes while suppressing detrimental long-term M1 responses. This strategic manipulation of the osteoimmune microenvironment throughout the healing timeline represents a critical mechanism underlying the enhanced bone regeneration observed in our experimental model, highlighting the therapeutic potential of biomaterial-mediated immunomodulation in regenerative medicine applications.

### 3.12 In vivo repair of femoral condyle defects in New Zealand rabbits using ICA@G/NHP composite materials

**Fig. 11:**
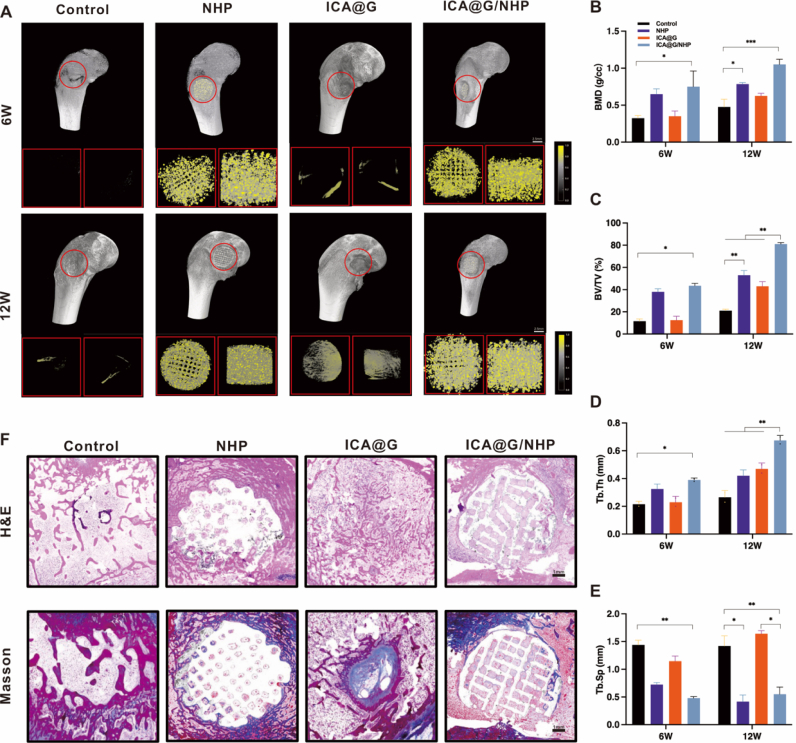
(A) Reconstructed 3D micro-CT images of femoral condyle defects in New Zealand rabbits at 6w and 12w. (B–E) Quantitative evaluation of bone regeneration parameters derived from micro-CT data, including bone mineral density (BMD), bone volume fraction (BV/TV), trabecular thickness (Tb.Th), and trabecular separation (Tb.Sp). (B) Representative histological analysis of the defect area at 12w via H&E and Masson staining, highlighting bone matrix remodeling and collagen deposition.

To evaluate the bone regenerative potential of ICA@G/NHP composite materials in a larger animal model, femoral condyle defects were created in New Zealand white rabbits. Adult New Zealand white rabbits (3.0–3.5 kg, n = 32) were randomly allocated into four experimental groups: control (untreated defect), NHP scaffold alone, ICA@G hydrogel alone, and ICA@G/NHP composite materials. Under general anesthesia, standardized cylindrical bone defects (6 mm diameter × 8 mm depth) were created in the lateral femoral condyle using a trephine drill with continuous saline irrigation. The defects were treated according to group assignment, with the control group receiving no intervention. Following thorough irrigation, the surgical sites were closed in anatomical layers. Micro-CT analysis was performed at 6 and 12 weeks post-surgery to assess bone regeneration. Three-dimensional reconstruction images (Fig. 11A) revealed progressive bone formation in all treatment groups, with the ICA@G/NHP composite demonstrating the most substantial new bone formation within the femoral condyle defects. Quantitative micro-CT analysis (Fig. 11B–E) showed significant differences between groups over time. At 6 weeks, bone mineral density (BMD) in the ICA@G/NHP group (0.65 ± 0.08 g/cm^3^) was significantly higher than the control (0.35 ± 0.05 g/cm^3^), NHP (0.68 ± 0.06 g/cm^3^), and ICA@G (0.40 ± 0.04 g/cm^3^) groups. This trend continued at 12 weeks, with the ICA@G/NHP group achieving BMD values of 1.05 ± 0.09 g/cm^3^, substantially exceeding all other groups. Bone volume fraction (BV/TV) analysis revealed similar patterns, with the ICA@G/NHP group showing 38.5 ± 3.2 % at 6 weeks and 78.2 ± 4.8 % at 12 weeks, significantly outperforming individual component groups and controls. Trabecular thickness (Tb.Th) measurements demonstrated enhanced bone architecture in the composite group, reaching 0.68 ± 0.05 mm at 12 weeks compared to 0.25 ± 0.03 mm in controls. Conversely, trabecular separation (Tb.Sp) was markedly reduced in the ICA@G/NHP group at both time points, indicating more mature and densely organized trabecular architecture. These quantitative parameters confirmed the superior osteogenic capacity of the composite scaffold in promoting effective bone defect repair. Histological examination at 12 weeks post-surgery revealed distinct regenerative patterns among treatment groups. H&E staining (Fig. 11F) demonstrated that the ICA@G/NHP group exhibited robust bone regeneration with well-organized trabecular bone structure and abundant osteocyte lacunae throughout the defect site. The regenerated bone showed mature lamellar organization with seamless integration into the surrounding host bone tissue. In contrast, the NHP group displayed moderate bone formation with predominantly woven bone architecture, while the ICA@G group showed limited and patchy bone regeneration with incomplete defect filling. The control group remained dominated by fibrous connective tissue with minimal evidence of bone formation. Masson's trichrome staining corroborated these findings, revealing dense, well-organized collagen matrix deposition in the ICA@G/NHP group that closely resembled native bone structure. The blue-stained collagen fibers demonstrated mature organization with proper mineralization patterns, indicating successful bone remodeling. Other treatment groups showed varying degrees of collagen organization, with the composite group clearly superior in terms of both quantity and quality of extracellular matrix formation. To investigate the immunomodulatory effects of the composite scaffold, immunofluorescence analysis was performed for macrophage polarization markers CD206 (M2 anti-inflammatory) and iNOS (M1 pro-inflammatory). At 12 weeks post-implantation, the ICA@G/NHP group demonstrated significantly enhanced CD206 expression throughout the defect area, indicating predominant M2 macrophage polarization. Strong green fluorescence signals were observed at the bone-scaffold interface and within newly formed bone tissue, suggesting a favorable anti-inflammatory microenvironment conducive to bone healing. Conversely, iNOS expression was markedly reduced in the ICA@G/NHP group, indicating suppression of pro-inflammatory M1 macrophage activation (Fig. S7).

The comprehensive results from the rabbit femoral condyle model demonstrated that ICA@G/NHP composite materials consistently promote enhanced bone regeneration through multiple mechanisms. The combination of the 3D-printed KNN/nHA/PLA scaffold with icariin-loaded gelatin methacryloyl hydrogel provided synergistic effects that not only accelerated bone healing but also modulated the immune microenvironment to create optimal conditions for tissue regeneration. The superior performance across micro-CT, histological, and immunological assessments confirms the clinical potential of this composite biomaterial strategy for treating large bone defects.

### 3.13 The molecular mechanism of ICA@G/NHP in promoting osteogenic differentiation of BMSCs

**Fig. 12:**
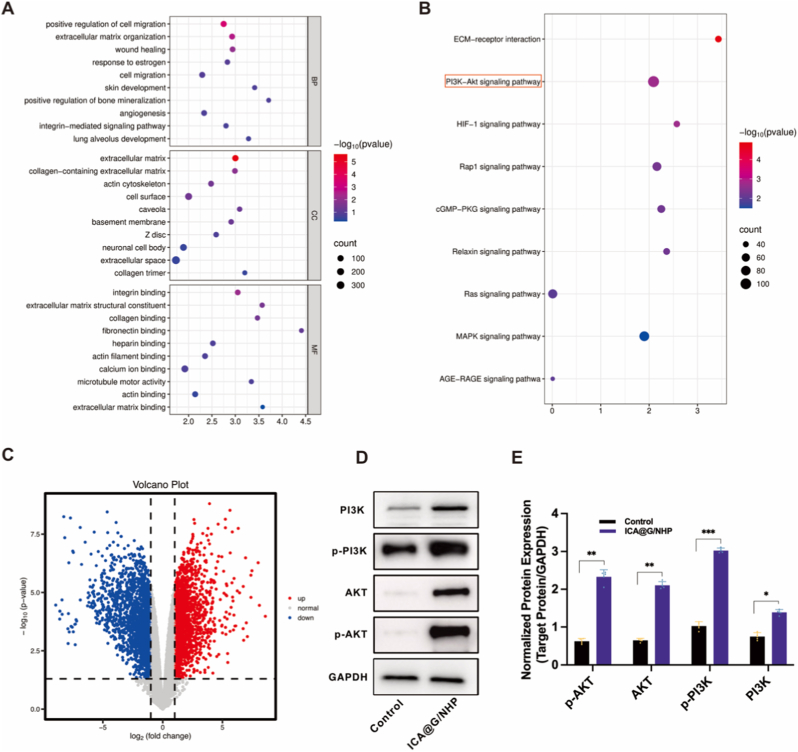
Molecular mechanism analysis of ICA@G/NHP-induced osteogenic differentiation. (A) Gene Ontology (GO) enrichment analysis showing biological processes (BP), cellular components (CC), and molecular functions (MF) significantly enriched in BMSCs cultured on ICA@G/NHP for 7 days. (B) KEGG pathway enrichment analysis revealing key signaling pathways activated by the composite scaffold. (C) Volcano plot displaying differentially expressed genes, with upregulated genes in red and downregulated genes in blue (fold change >1.5, p < 0.05). (D) Western blot analysis of PI3K/Akt signaling pathway proteins in BMSCs cultured on control and ICA@G/NHP scaffolds. (E) Quantitative analysis of protein expression levels normalized to GAPDH. Data are presented as mean ± SD (n = 3). ∗p < 0.05, ∗∗p < 0.01, ∗∗∗p < 0.001 vs. control group.

To elucidate the molecular mechanisms underlying the enhanced osteogenic differentiation induced by ICA@G/NHP, we performed RNA-seq analysis of BMSCs cultured on the composite scaffold for 7 days. Gene Ontology (GO) enrichment analysis revealed significant upregulation of biological processes related to cellular mechanotransduction and osteogenesis (Fig. 12A). The most significantly enriched terms included positive regulation of cell migration, extracellular matrix organization, wound healing response, and integrin-mediated signaling pathway, indicating that ICA@G/NHP activates mechanosensitive cellular processes crucial for bone formation.KEGG pathway enrichment analysis further identified key signaling cascades activated by the piezoelectric scaffold (Fig. 12B). Notably, the ECM-receptor interaction pathway showed the highest significance, followed by the PI3K-Akt signaling pathway, which is known to play a central role in mechanotransduction and osteogenic differentiation. Other significantly enriched pathways included HIF-1 signaling, Rap1 signaling, and MAPK signaling pathways, all of which are involved in cellular responses to mechanical stimuli and bone formation processes.Volcano plot analysis revealed 1247 differentially expressed genes (DEGs) in BMSCs cultured on ICA@G/NHP compared to controls, with 623 upregulated and 624 downregulated genes (Fig. 12C). The upregulated genes were predominantly associated with mechanosensitive pathways, calcium signaling, and osteogenic differentiation markers.To validate the transcriptomic findings, we examined the activation of the PI3K/Akt signaling pathway, which emerged as one of the most significantly enriched pathways. Western blot analysis demonstrated substantial upregulation of both PI3K and phosphorylated PI3K (p-PI3K) in BMSCs cultured on ICA@G/NHP compared to controls (Fig. 12D and E). Similarly, both total Akt and phosphorylated Akt (p-Akt) levels were significantly increased, confirming the activation of this critical mechanotransduction pathway. The enhanced phosphorylation of PI3K and Akt suggests that the piezoelectric properties of the KNN component in the scaffold generate electrical signals that activate mechanosensitive ion channels and downstream signaling cascades, ultimately promoting osteogenic differentiation of BMSCs.

## 4 Conclusion

The present study proposes an ICA@G/NHP composite scaffold system integrating piezoelectricity that synergistically promotes bone regeneration and immunomodulation, achieving sequential osteogenesis-immunomodulation coupling for regenerative processes. The 3D-printed scaffold exhibited ordered interconnected porous architecture, stable piezoelectric output, and appropriate icariin release kinetics that align with early bone healing demands. In vitro assessments demonstrated that ultrasonic activation was essential to elicit the piezoelectric effect of the scaffold, which enhanced BMSCs osteogenic differentiation. Furthermore, the incorporated ICA component effectively promoted endothelial cell migration and M2 macrophage polarization, resulting in a synergistic osteogenic outcome. In vivo validation in rabbit femoral condyle and rat cranial defect models confirmed superior bone regeneration with enhanced M2/M1 macrophage ratios and mature bone architecture formation.Mechanistically, RNA transcriptomic analysis identified PI3K/Akt as the central signaling pathway, with Western blot validation confirming phosphorylation activation of PI3K and Akt. Network pharmacology analysis further elucidated the molecular mechanisms by which icariin creates a pro-regenerative microenvironment through regulation of macrophage polarization. The integration of ultrasound-triggered piezoelectric cues with temporally controlled drug release has been demonstrated to enable composite functional scaffolds to adapt to complex tissue reconstruction, thereby overcoming spatiotemporal decoupling challenges in bone repair.This work represents a significant advancement in the field of intelligent scaffold design for critical-sized bone defects, with particular emphasis on microenvironment-adaptive regeneration strategies. By integrating piezoelectric stimulation, immunomodulation, and osteoinduction within a single platform, the ICA@G/NHP system provides an innovative alternative to conventional bone repair approaches, addressing clinical challenges including donor site morbidity associated with autografts, immune rejection risks of allografts, and the lack of active immunomodulatory capacity in existing biomaterials. Despite the promising preclinical outcomes, successful clinical translation requires addressing several critical challenges. Scale-up manufacturing must ensure consistent KNN distribution and piezoelectric properties across larger constructs while maintaining quality control standards for batch-to-batch reproducibility. Sterilization protocols need optimization to preserve both piezoelectric functionality and icariin bioactivity, as conventional methods may compromise material performance. The composite device nature necessitates regulatory approval as a combination product under FDA guidelines, requiring comprehensive biocompatibility studies and validation of ultrasound activation protocols. Cost-effectiveness analysis must demonstrate economic viability compared to current standard care, considering potential long-term savings from reduced complications and improved outcomes. Future research should focus on optimizing activation protocols for specific anatomical applications, developing patient-specific designs, and establishing clinical-grade manufacturing standards to advance this immunomodulatory platform toward therapeutic application.

## CRediT authorship contribution statement

**Yongbin Wang:** Writing – review & editing, Writing – original draft, Methodology, Investigation, Formal analysis, Data curation, Conceptualization. **Han Zhang:** Methodology, Investigation. **Zhili Xu:** Writing – original draft, Validation, Supervision. **Weihang Zhu:** Project administration. **Sheng Chang:** Software. **Jiahao Wei:** Data curation. **Shuqing Chen:** Investigation. **Yong Liu:** Methodology. **Weiqing Kong:** Writing – review & editing, Resources. **Jianwei Guo:** Writing – review & editing, Writing – original draft, Funding acquisition, Conceptualization.

## Declaration of competing interest

The authors declare that they have no known competing financial interests or personal relationships that could have appeared to influence the work reported in this paper.

## Data Availability

Data will be made available on request.
