# Supplementary material for: Icariin-loaded GelMa hydrogel encapsulated potassium sodium niobate biomimetic piezoelectric scaffold regulates macrophage polarization to accelerate bone defect repair
Source: Mater Today Bio. 2025 Oct 30;35:102476. doi: 10.1016/j.mtbio.2025.102476 (PMC12639499; doi:10.1016/j.mtbio.2025.102476)
Supplement: Multimedia component 1 [file mmc1.docx]

**Supplementary Information**

**Icariin-loaded Gemal Hydrogel Encapsulated Potassium Sodium Niobate Biomimetic Piezoelectric Scaffold Regulates Macrophage Polarization** **to Accelerate Bone Defect Repair**

**Authors:**

Yongbin Wang^#^, Han Zhang^#^, Zhili Xu^#^, Weihang Zhu, Sheng Chang, Jiahao Wei, Shuqing Chen, Yong Liu*, Weiqing Kong*, Jianwei Guo *

**Affiliations:**

Yongbin Wang, Han Zhang, Weihang Zhu, Sheng Chang, Jiahao Wei, Shuqing Chen, Yong Liu, Jianwei Guo

Department of Orthopedics, the Affiliated Hospital of Qingdao University, Qingdao, 266003, People’s Republic of China

Zhili Xu

School of Anesthesiology, Shandong Second Medical University, Weifang, Shandong, 261053，People’s Republic of China

Weiqing Kong

Department of Orthopedic Surgery，Xuzhou Central Hospital, Xuzhou Clinical School of Xuzhou Medical University,199 Jiefang South Road,Xuzhou,221009, People’s Republic of China

***Corresponding author:**

Dr. Jianwei Guo

Department of Orthopedics, the Affiliated Hospital of Qingdao University, Qingdao, 266003, People’s Republic of China

E-mail: guojw_qdfy@163.com

Dr. Weiqing Kong

Department of Orthopedic Surgery，Xuzhou Central Hospital, Xuzhou Clinical School of Xuzhou Medical University,199 Jiefang South Road,Xuzhou,221009, People’s Republic of China

E-mail: wqingkong@163.com

Dr. Yong Liu

Department of Orthopedics, the Affiliated Hospital of Qingdao University, Qingdao, 266003, People’s Republic of China

E-mail: [liuyongdr20@163.com](mailto:liuyongdr20@163.com)

**Supplementary Figures:**


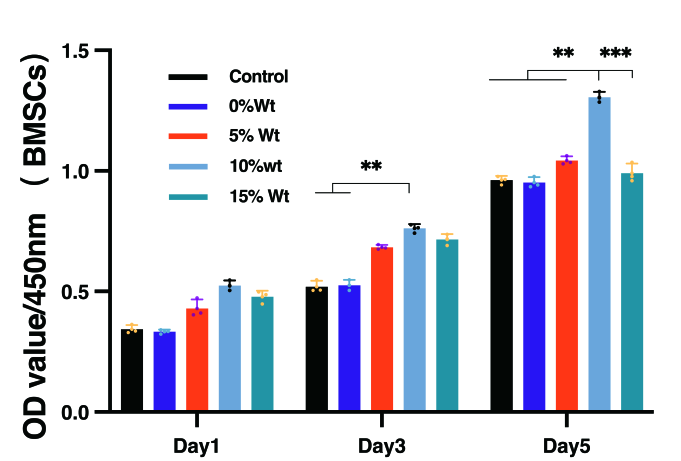


**Supplementary Figure S1.** The CCK-8 assay was utilized to evaluate the effects of NHP scaffolds with varying KNN content on the viability of bone marrow mesenchymal stem cells (BMSCs) on days 1, 3, and 5.

**
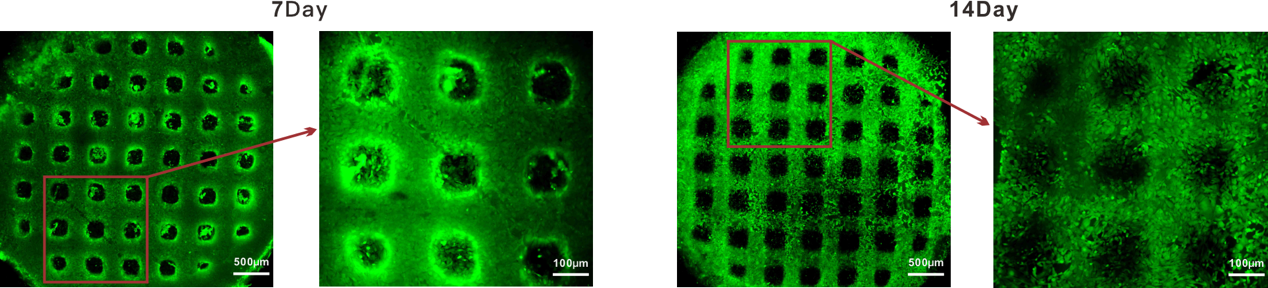
**

**Supplementary Figure S2.C**ellular morphology of BMSCs seeded on NHP scaffolds on days 7 and 14.


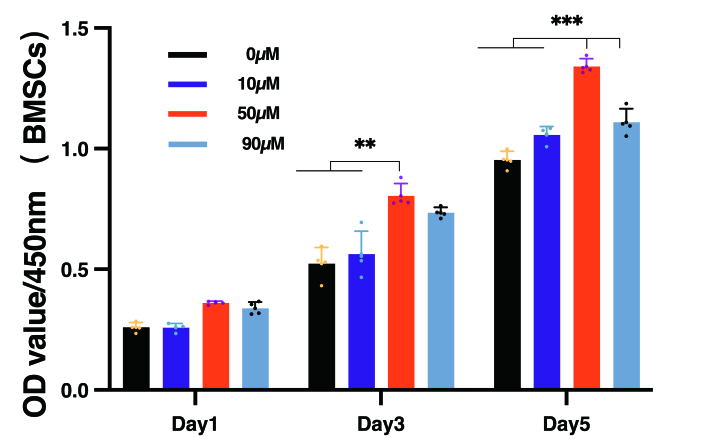


**Supplementary Figure S3.** The CCK-8 assay was utilized to evaluate the effects of varying ICA concentrations on the viability of bone marrow mesenchymal stem cells (BMSCs) on days 1, 3, and 5.


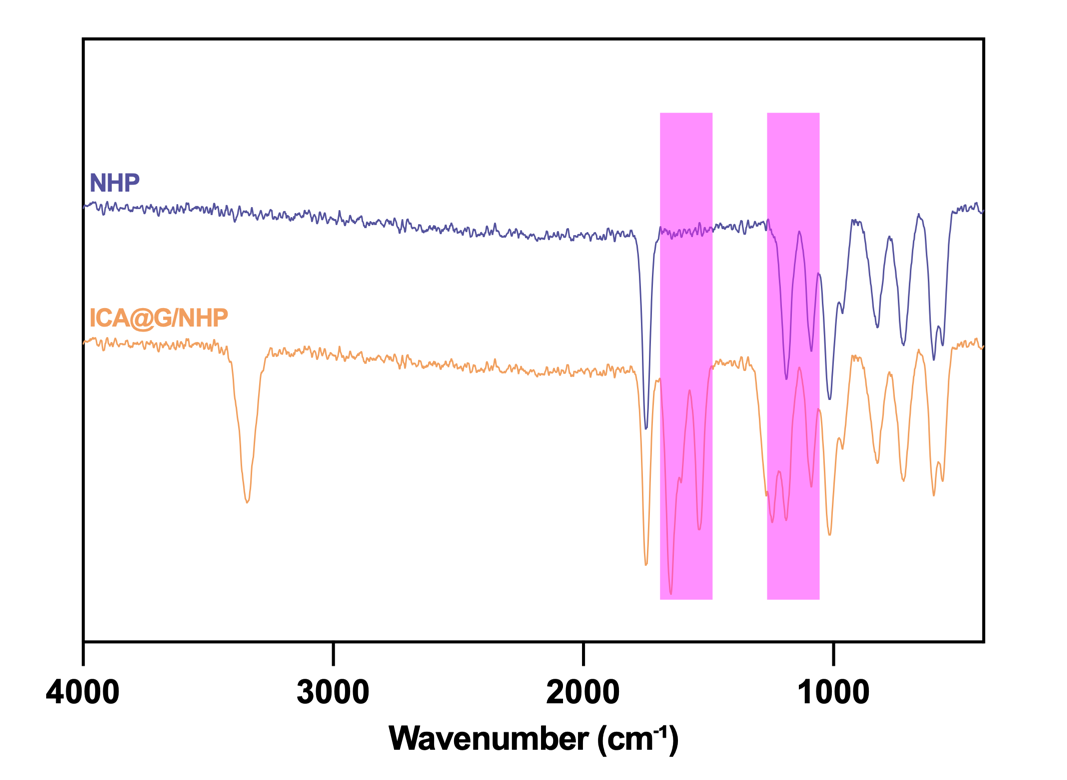


**Supplementary Figure S4.** FTIR spectra of the scaffolds.


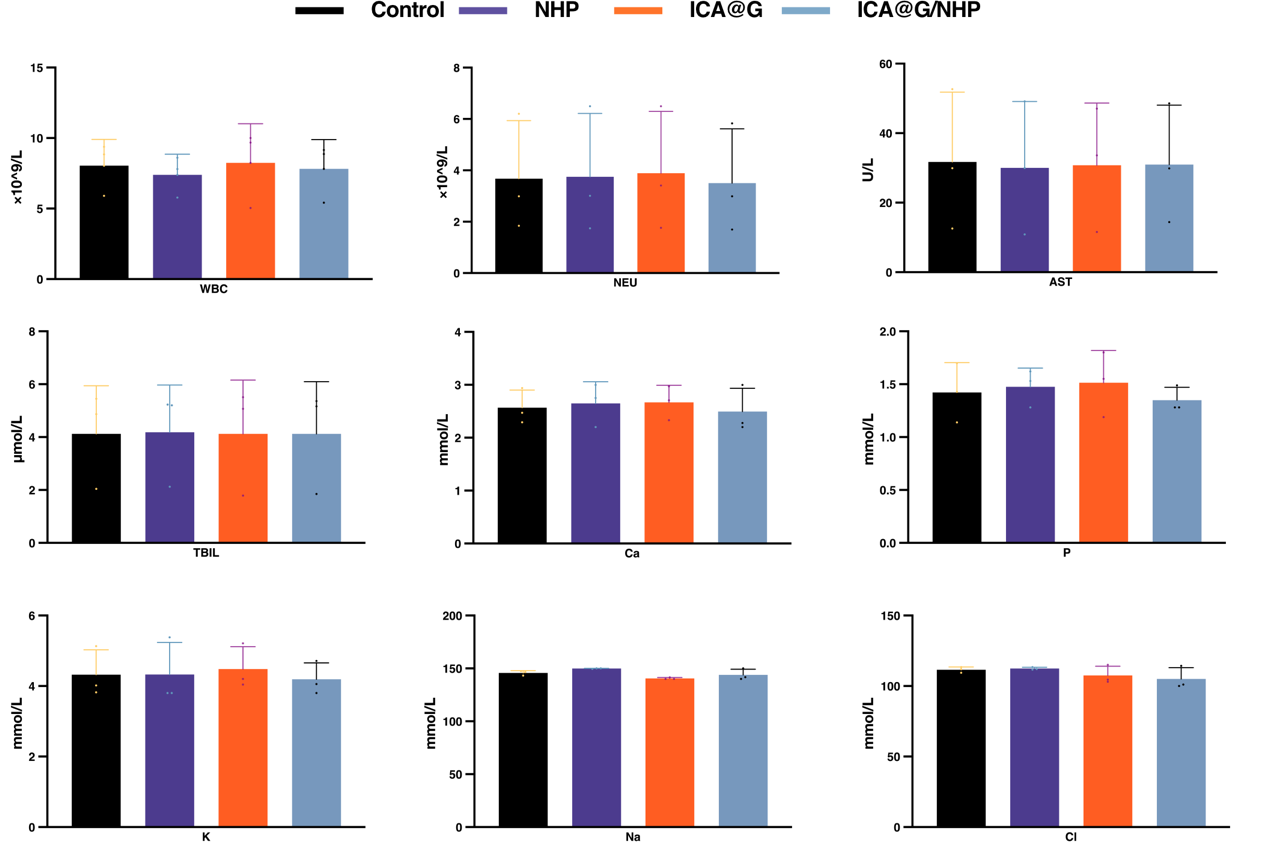


**Supplementary Figure S5.** C**omprehensive blood chemistry analysis.**


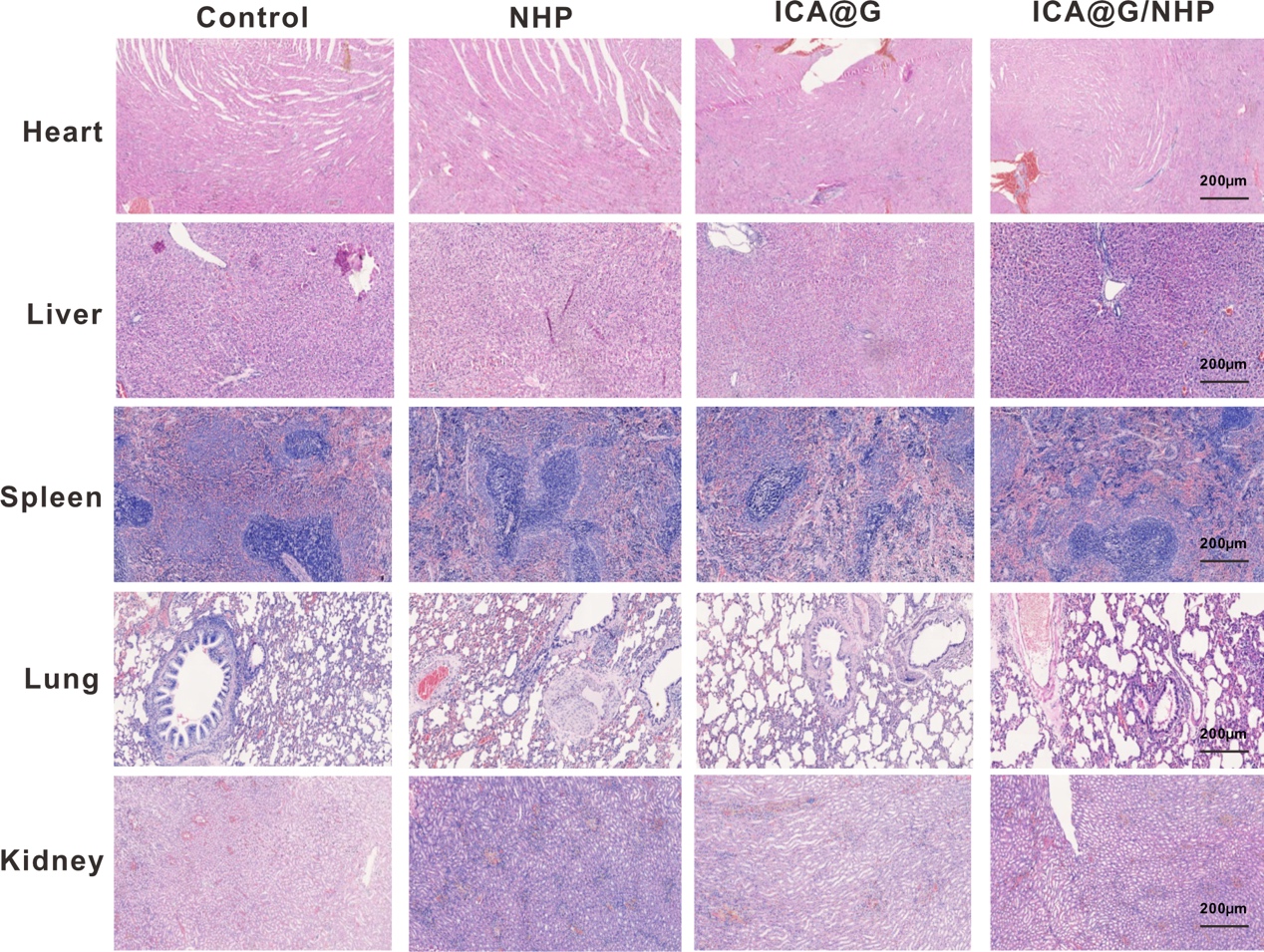


**Supplementary Figure S6.** Hematoxylin-eosin staining analysis of major organs in rats sacrificed in 12 weeks after material implantation.


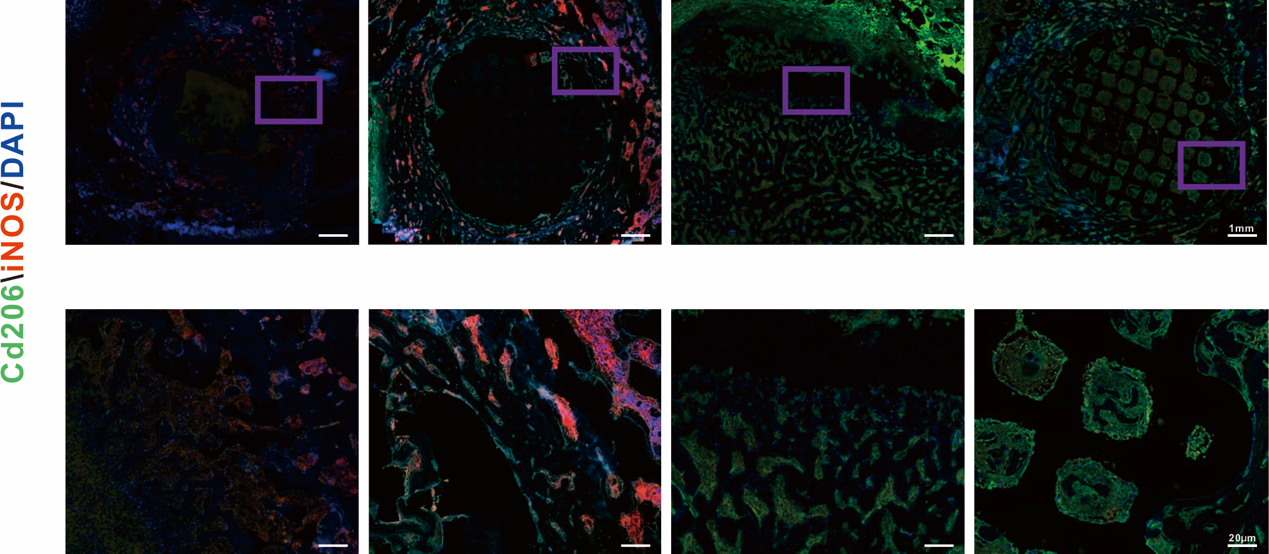


**Supplementary Figure S7.** IF for CD206 and iNOS at the 12-week time point.

**Supplementary Tables:**

**Table S1. Primer sequences of each osteogenesis-related gene**

| Gene | Direction | Sequence (5’-3’) |
| --- | --- | --- |
| ALP | Forward | GCCTACTTGTGTGGCGTGAA |
|  | Reverse | AGGATGGACGTGACCTCGTT |
| RUNX2 | Forward | TCAAGGGAATAGAGGGGATGC |
|  | Reverse | GGGAGGACAGAGGGAACCACC |
| BMP2 | Forward | CGGAAGCGTCTTAAGTCCAG |
|  | Reverse | CATGCCTTAGGGATTTTGGA |
| COL1 | Forward | GACATGTTCAGCTTTGTGGACCTC |
|  | Reverse | GGGACCTTAGGCCATTGTGTA |
| OPN | Forward | GGGATCGCTGAAGGCATAAA |
|  | Reverse | CAGCCGTAAAAGGCTTAAAA |
| OCN | Forward | GGACCTCTCTCTCTGCTCACTCTG |
|  | Reverse | ACCTTACTGCCCTCCTGCTTGG |
| GAPDH | Forward | AGAAGGCTGGGGCTCATTTG |
|  | Reverse | AGGGGCCATCCACAGTCTTC |

**Table S2. Primer sequences of each angiogenesis-related gene**

| Gene | Direction | Sequence (5’-3’) |
| --- | --- | --- |
| **vWF** | Forward | GGAACCTGACCAAGGACAGC |
|  | Reverse | CTGGGCTTCACAGTCAGGT |
| **CD31** | Forward | CCAGGTGGTGAAGATGATGC |
|  | Reverse | GGTGGTGGCTGTAGTCATCC |
| **ANG** | Forward | CCTGGGACTTCGCAACTAC |
|  | Reverse | TGCCTTCAGCTTCACAGTCC |
| **β-actin** | Forward | CATGTACGTTGCTATCCAGGC |
|  | Reverse | CTCCTTAATGTCACGCACGAT |

**Table S3. Primer sequences of each macrophage polarization-related gene**

| Gene | Direction | Sequence (5'-3') |
| --- | --- | --- |
| TNF-α | Forward | CCTGTAGCCCACGTCGTAGC |
|  | Reverse | TTGAGATCCATGCCGTTGGC |
| iNOS | Forward | GTTCTCAGCCCAACAATACAAGA |
|  | Reverse | GTGGACGGGTCGATGTCAC |
| Arg1 | Forward | CTCCAAGCCAAAGTCCTTAGAG |
|  | Reverse | AGGAGCTGTCATTAGGGACATC |
| CD206 | Forward | CTCTGTTCAGCTATTGGACGC |
|  | Reverse | CGGAATTTCTGGGATTCAGCTTC |
| GAPDH | Forward | AGAAGGCTGGGGCTCATTTG |
|  | Reverse | AGGGGCCATCCACAGTCTTC |
